# Validity of the Diet Quality Questionnaire Compared with Observed Intake for Estimating Population-Level Diet Quality in Rwandan Adults

**DOI:** 10.1016/j.cdnut.2025.107628

**Published:** 2025-12-26

**Authors:** Betül TM Uyar, Inge D Brouwer, Anna W Herforth, Rhys Manners, Maria Giovanna Delfine, Rosil Hesen, Karin J Borgonjen-van den Berg, Edith JM Feskens, Elise F Talsma

**Affiliations:** 1 Division of Human Nutrition and Health, Wageningen University and Research, the Netherlands; 2 International Food Policy Research Institute, Washington DC, United States; 3 International Institute of Tropical Agriculture, Kigali, Rwanda

**Keywords:** DQQ, MDD-W, weighed food record, 24-h recall, dietary assessment, nutrition surveillance, method of triads, validation

## Abstract

**Background:**

The diet quality questionnaire (DQQ) is a standardized low-burden tool for collecting data on minimum dietary diversity for women (MDD-W) and other population-level diet quality indicators related to risk of noncommunicable disease (NCD). Although 24-h recalls (24hRs) are often used for evaluating validity of DQQ, they may underestimate consumption of specific food groups. Therefore, comparison with observed weighed food records (OWFR), can provide a more accurate assessment of DQQ criterion validity.

**Objective:**

The aim of this study is to evaluate criterion validity of DQQ for estimating population-level diet quality using OWFR and 24hR as reference methods.

**Methods:**

Cross-sectional data were collected among 281 Rwandan adults (Musanze district), using OWFR, DQQ, and 24hR on the same day. Diet quality indicators derived from each method were compared using parametric and nonparametric methods and the method of triads, which calculates pairwise validity coefficients to evaluate accuracy (low: <0.30; moderate: 0.30–0.70; high >0.70).

**Results:**

Mean percent agreement in food group consumption data was high: 93% (DQQ-OWFR; DQQ-24hR). Compared with OWFR, DQQ overestimated MDD-W-prevalence [DQQ: 46.0% compared with OWFR: 40.4%; +6 percentage points (pp), *P* > 0.05], whereas using 24hR, the MDD-W-prevalence was 29.8% (16.2 pp < DQQ, *P* < 0.05, and 10.6 pp < OWFR, *P* < 0.05). Compared with OWFR, mean scores of food group diversity score (FGDS) and NCD-Protect were 0.2 (ns) and 0.2 (*P* = 0.01) points higher by DQQ, respectively, and 0.4 (*P* < 0.001) points higher by DQQ compared with 24hR. NCD-Risk median scores were 0 across methods. For DQQ, validity coefficients were 0.70 (FGDS), 0.67 (NCD-Protect), and 0.66 (NCD-Risk), compared with 0.93, 0.89, and 0.59 for OWFR, respectively, and 0.84, 0.83, and 0.98 for 24hR, respectively.

**Conclusions:**

The DQQ showed high agreement with OWFR and 24hR for collecting population-level food group consumption data, and slight overestimations of diet quality indicator scores compared with observed intakes. DQQ is a valid and practical method for collecting population-level food group consumption data and estimating diet quality.

## Introduction

Population-level dietary intake of people can be measured by several assessment methods such as self-report 24-h recalls (24hR) and observed weighed food records (OWFR) [1,2]. These methods, however, are often infeasible to implement at a large scale, especially in resource-limited low- and middle-income countries (LMICs), because they are relatively expensive to administer due to extensive training required for enumerators as well as the substantial time and expertise needed for data processing [2]. These methods are therefore not feasible for routine diet quality monitoring at population level.

The Diet Quality Questionnaire (DQQ) is a survey module designed for monitoring indicators of diet quality, to collect valid population-level food group consumption data at low cost and low burden for respondents, enumerators, and analysts [3,4]. The tool has been adapted and implemented in >90 countries, in the Gallup World Poll and other country-owned surveys, including the Demographic and Health Surveys, for measuring diet quality at population level [[4], [5], [6]]. The DQQ is a list-based dietary assessment method asking about consumption of 29 food groups in the previous day and night. In a 3-LMIC validation study of DQQ against 24hR among females, the 2 methods produced small differences between population-level diet quality scores, with a tendency toward higher reporting of consumption of several food groups including fruits, vegetables, and sweets in the DQQ [3,7]. Because both DQQ and 24hR rely on memory to recall dietary intake, it is difficult to assess which method captures diets more accurately. Although the 24hR was used as a reference method, it is possible that respondents might have correctly reported consumption of these food groups in the DQQ but might have forgotten to recall them when not specifically probed for during the 24hR [3,7]. Previous studies in low-income countries comparing 24hR to OWFR also found that errors of underreporting are more frequent than overreporting in 24hR, especially with fruit, snacks, and beverages being underreported more frequently than foods typically consumed in main meals [[8], [9], [10]]. Therefore, further insights about accuracy of the DQQ can be gained by comparison to a method that does not rely on memory such as observed OWFR [2]. Assessing whether the DQQ produces data that are comparable with data from a reference method (e.g. OWFR) is also known as criterion validity [11]. In addition, the method of triads is a triangular comparison between dietary assessment methods that estimates correlations between each type of method and latent true (but unknown) dietary intake, which is used for the validation of dietary assessment methods for accurately estimating dietary intake [12].

This study aimed to investigate criterion validity of DQQ for collecting population-level food group consumption data and estimating diet quality against both OWFR and 24hR as reference methods among adults in Rwanda, within the framework of a larger study piloting a data collection system for high-frequency diet quality monitoring based on administration of the DQQ via mobile phone [13].

## Methods

### Study population

Cross-sectional data were collected among *n* = 308 females and males aged ≥18 y in Musanze, a district in the Northern province of Rwanda, each from a different household, during a period of 4 wk in July 2023. More than 3000 individuals who voluntarily participated in a previous pilot study were contacted by VIAMO (www.viamo.io) via an interactive voice response messaging system [13]. Consenting respondents were randomly assigned to an ID. Participation was confirmed 24 h before the start of the data collection with each respondent [14].

### Data collection

Sociodemographic data such as age, education level, household size, and dietary data were collected by trained data collectors using questionnaires translated into the local language (Kinyarwanda). Anthropometric data (height and weight) were collected using standardized procedures of the WHO [15]. All data were collected on tablets using the KoboToolbox [16] and Open Data Kit–based forms for sociodemographic, anthropometric, DQQ, and OWFR data, and the web-based Catch-24 app for 24hR data.

### Dietary assessment tools

In this study, 3 dietary assessment tools were used among all respondents: the OWFR, DQQ, and multi-pass 24hR, for collecting food group consumption data. On day 1 the OWFR was administered, and the next day (day 2) the DQQ was administered followed by the 24hR.

#### Observed weighed food record

For the OWFR, respondents were shadowed by enumerators from 06:00 to 20:00. Enumerators recorded and weighed all food and drinks consumed by the respondents to the nearest decimal in g using a digital kitchen scale with a precision of 1 g (SF-400). When mixed dishes were consumed, enumerators recorded the empty pot weight, all ingredients used, the weight of the pot containing the prepared dish, the empty plate, the portion on the plate, and leftovers. For recipes consumed away from home, enumerators followed respondents and engaged with the cooks to document ingredients and weigh each ingredient when feasible. Responses were supplemented by 24hR to identify any food or drink that may have been consumed before 06:00 and after 20:00 (i.e. we pulled these items from 24hR into OWFR for completing the day, because enumerators could not stay in households for 24 h). A food composition table (FCT) was developed by incorporating the energy and nutrient content of each food item consumed in both OWFR and 24hR, using FCTs from other countries [[17], [18], [19]]. For both OWFR and 24hR, energy and nutrient intakes were calculated using Compl-eat (Wageningen University and Research, Version 2.0).

#### Diet quality questionnaire

The Rwanda-adapted national DQQ [20] was administered either in person (enumerator-DQQ) or on mobile phone through self-administration (mobile-DQQ), with participants randomly assigned to the mode of administration, as described in detail in Manners et al. [14] (see Supplementary material for the Rwanda-adapted DQQ used in our study). For the enumerator-DQQ, during data collection, enumerators administered the DQQ by reading aloud the list-based questions comprising 29 food groups to the respondents without any probing or additional dialogue [4]. For the mobile-DQQ, respondents received the DQQ via unstructured supplementary service data—an Short Message Service (SMS)-like system—message by 06:00 on day 2. After completing each DQQ-question, the answers were automatically sent to the server of VIAMO [13]. Mobile-DQQ data were cleaned according to the quality control method described by Manners et al. [14], resulting in the exclusion of 7 respondents. In addition, 20 respondents were excluded, of whom 3 did not have a DQQ submitted, 7 had enumerator errors (where enumerators had failed to adhere to the data collection protocols), and 10 whose age and sex based on DQQ and OWFR did not match. Subsequently, a total of *n* = 281 observations (*n* = 154 enumerator-DQQ, and *n* = 127 mobile-DQQ) were left to be analyzed. Manners et al. [14] showed enumerator- and mobile-DQQ groups were comparable, with only minor differences [14].

#### Multi-pass 24hR

For the multi-pass interviewer-administered 24hR, respondents were not allowed to look at their OWFR from the day before. During 24hR, respondents were asked to name all food and drinks consumed including ingredients and cooking methods of mixed dishes between waking up and sleeping the preceding day [21]. Respondents were also asked to estimate the weight of foods, drinks, and ingredients consumed. For food items available in households, amounts of all foods, drinks, and ingredients of mixed dishes consumed were weighed to the nearest decimal in grams using a digital kitchen scale (SF-400) with a precision of 1 g. When food items were not available for direct measurements, substitutes such as water or maize flour were weighed and converted using conversion factors (weight-to-weight, volume-to-weight, standard serving sizes, and waste factors). Conversion factors and waste factors were collected during a market survey in 3 different markets and local restaurants in the Musanze district. For mixed dishes consumed out of home, cooks of 3 different market stalls or shops were asked to recall their preparation, including types and amounts of all ingredients used, volumes (i.e. how much was prepared with the recalled ingredients), and amounts of portions that could be served out of the total prepared dish. Mean amounts per ingredient of the 3 recipes were calculated for standardized mixed dishes.

### Data analysis

#### Food group variables construction

Foods and drinks originating from OWFR and 24hR were coded into the 29 food groups of the DQQ using its food group classification guide [50]. Population-level food group consumption prevalence and diet quality indicator outcomes were presented using regular procedures according to the minimum dietary diversity for women (MDD-W) measurement guide of excluding foods consumed in amounts <15 g in OWFR and 24hR to avoid inflating FGDSs [6,23]. We used context-specific exceptions to the 15 g cutoff, on the principle of including items that were important to micronutrient intakes even in small amounts [24]. For this, we examined micronutrient contribution of dried foods to the total diet, and found that 7 g dried small fish contributed 100% of the recommended nutrient intake for vitamin B12 (2.4 μg) [25] (median intake of 7 g, IQR 4–15 g) (Supplemental Table 1). Small dried fish was therefore retained in analyses regardless of amounts consumed. For analyses excluding <15 g of dried small fish in OWFR and 24hR see Supplemental Tables 2a and b). No other items were identified that had important micronutrient contributions when consumed in small amounts. Of note, a similar conclusion was reached in the process of adapting the DQQ based on consultation with country-specific key informants [6], who recommended that dried small fish were an important food that was not used just to add flavor. Dried small fish were also included in other East African and some Southern African countries [26].

#### Food group consumption

Food group consumption was presented in prevalence (%) based on OWFR, DQQ, and 24hR. Differences in population-prevalence were calculated by the food group consumption prevalence from DQQ minus OWFR (DQQ-OWFR), DQQ minus 24hR (DQQ-24hR), and 24hR minus OWFR (24hR-OWFR, Supplemental Tables 3-6) and compared for statistical differences using McNemar’s test (*P* < 0.05). Mean difference in population-consumption–prevalence of all food groups was calculated by summing the difference of each food group and dividing it by the total number of food groups. Percent agreement (PA) computed from cross tabulations was used to evaluate measurement agreement between pairwise comparisons of methods of DQQ and OWFR, DQQ and 24hR, and 24hR and OWFR, for consumption of food groups. Misreporting [false positives (FP) and false negatives (FN)] of food group consumption was examined using cross tabulations. The FP rate (type 1 error) was the percentage of respondents who reported having consumed a food group based on DQQ, but not having consumed it based on reference methods OWFR or 24hR (i.e. overreporting). The FN rate (type 2 error) was the percentage of respondents not having consumed a food group based on DQQ, but having consumed it based on reference methods OWFR or 24hR (i.e. underreporting). In addition to testing prevalence-differences between methods for statistical significance, we considered whether observed differences as well as observed FP and FN rates were meaningful in terms of public health significance using a cutoff point of >10 percentage points (pp) between tools [3,[27], [28], [29]], which is conservative when compared with cutoffs of 15 or 20% used in evaluating validity of dietary assessment methods for accurately reporting energy and nutrient intake [30,31].

#### Diet quality indicators

Diet quality indicators were constructed based on the 29 food groups, originating from DQQ, 24hR, or OWFR. We used indicators (potentially) useful for global monitoring of diets at the population level. Binary indicators used in this study are the MDD-W [23], All-5 [32], the protective food consumption (PFC), and unhealthy food consumption (UFC) [33]. Continuous indicators used were the food group diversity score (FGDS) [34], noncommunicable disease (NCD)-Protect and NCD-Risk [27]. Binary indicators were analyzed in the same way as food group consumption analysis, i.e. using McNemar’s test, PA, FP, and FN. Continuous indicators were checked for normality using Q–Q plots. Means (SD) were calculated for normally distributed indicators, and median (P^25^–P^75^) for non-normal distributed indicators. All continuous indicators were tested for statistically significant differences (*P* < 0.05) using the paired samples *t*-test for normally distributed indicators, and the Wilcoxon signed-rank test for non-normally distributed indicators. A difference in means or medians between DQQ and 24hR or OWFR exceeding 10 pp was considered practically important [[27], [28], [29]].

#### Method of triads to compare the OWFR, DQQ, and 24hR for accurately estimating diet quality indicators

**FIGURE 1:**
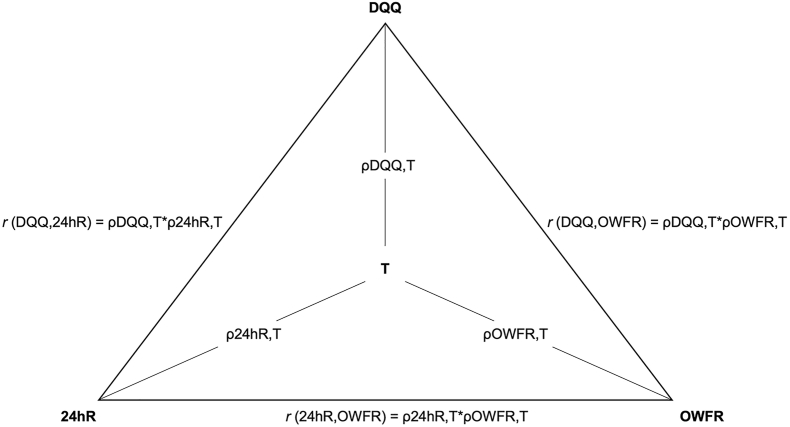
Method of triads to estimate true diet quality, using diet quality indicator scores derived from DQQ, 24hR, and OWFR adapted from Figure 1 of Kaaks (1997) [12]. 24hR, diet quality indicator score calculated from multi-pass 24-h open recall; DQQ, diet quality indicator score calculated from Diet Quality Questionnaire; OWFR, diet quality indicator score calculated from observed weighed food record; r, Pearson correlation coefficient between the diet quality indicator score calculated between DQQ / 24hR/ OWFR; T, true but unknown diet quality indicator score; ρ, validity coefficient of diet quality indicator score calculated from DQQ / 24hR/ OWFR in relation to T.

The method of triads (Figure 1) was used to evaluate all 3 dietary assessment methods (OWFR, DQQ, and 24hR) for accuracy in estimating continuous diet quality indicator scores in relation to true (T) but unknown diet quality by calculating validity coefficients (ρ) through using correlation coefficients (*r*) between the 3 dietary assessment methods. True diet quality must be considered as values of latent variables, which reflect reality, but which cannot be measured without error [2,35]. To calculate validity coefficients, first, the Pearson’s correlation coefficient (*r*) was determined between *1*) DQQ and OWFR (DQQ-OWFR), *2*) DQQ and 24hR (DQQ-24hR), and *3*) 24hR and OWFR (24hR-OWFR) of each diet quality indicator score. Thereafter, validity coefficients of each indicator derived from each method (DQQ, 24hR, and OWFR) in relation to the estimated T diet quality indicator scores were calculated using the following formulas [35]:

$$\rho \text{DQQ},T=\sqrt{\left[\left(r\left(DQQ,OWFR\right)\times r\left(24hR,DQQ\right)\right)\;/\;r\left(24hR,OWFR\right)\right]}$$

$$\rho 24\text{hR},T=\sqrt{\left[\left(r\left(24hR,OWFR\right)\times r\left(24hR,DQQ\right)\right)/\;r\left(DQQ,OWFR\right)\right]}$$

$$\rho \text{OWFR},T=\sqrt{\left[\left(r\left(DQQ,OWFR\right)\times r\left(24hR,OWFR\right)\right)/\;r\left(24hR,DQQ\right)\right]}\text{.}$$

Validity coefficients range from 0 to 1 and are classified as low: <0.30 (indicating low performance of the dietary assessment method for accurately estimating diet quality); moderate: 0.30 to 0.70 (indicating moderate performance of the dietary assessment method for accurately estimating diet quality); and high: >0.70 to 1 (indicating high performance of the dietary assessment method for accurately estimating diet quality) [36].

All analyses were performed for the total study population of adults and separately for females and males (Supplemental Tables 7-11). SPSS version 29.0 (IBM Corporation) was used for data analysis using a *P* value of < 0.05.

## Results

Table 1 describes participant characteristics. More than half (58%) of the study sample were female, 40% of respondents were aged between 25 and 34 y, and 74% of adults had a normal weight. Primary education was the highest level of education attained for 55% of respondents. About 72% of respondents were partnered (either married or nonmarried). Household size was about 5 people, and nearly 70% of the study population resided in rural areas, with 63% belonging to poor economic groups (Table 1) [37,38].

**TABLE 1:** Sociodemographic and anthropometric characteristics of adults in Northern Rwanda

Characteristic	*n* = 281
Sex [n (%)]	
Females	161 (57.3)
Males	120 (42.7)
Age (y) [*n* (%)]	
18–24	46 (16.4)
25–34	111 (39.5)
35–44	77 (27.4)
> 45	47 (16.7)
BMI^1^ (kg/m^2^), median (25th–75th percentiles) [*n* (%)]	23.2 (21.4–25.0)
Underweight^2^	3 (1.1)
Normal weight^2^	202 (73.7)
Overweight or obese^2^	69 (25.2)
Residential area [*n* (%)]	
Rural	188 (66.9)
Peri-urban	77 (27.4)
Urban	16 (5.7)
Economic group (Ubudehe) 3 [*n* (%)]	
1	27 (9.7)
2	147 (53.1)
3	103 (37.2)
Education^4^, highest level completed [*n* (%)]	
No (formal) education	32 (11.6)
Primary education	153 (55.2)
Secondary or higher^5^ education	92 (33.2)
Relationship status^6^ [*n* (%)]	
Single	78 (27.8)
Partnered	202 (71.9)
Household size [mean (SD)]	4.6 (1.6)
Religion [*n* (%)]	
Christianity (Catholic)	108 (38.4)
Christianity (Protestant)	143 (50.9)
Other	30 (10.7)

1 *n* = 274 without pregnant females (*n* = 6) and missing data (*n* = 1).

2 Weight status was based on BMI according to the WHO recommendations [15]: <18.5 kg/m^2^ for underweight, ≥18.5–24.9 kg/m^2^ for normal weight, ≥25.0–29.9 kg/m^2^ for overweight, and ≥30.0 kg/m^2^ for respondents with obesity.

3 *n* = 277 without missing data (*n* = 4), Ubudehe is based on the classification of Rwanda’s Government with group 1 and 2 for “poorest” households, with group 1 comprising very poor and vulnerable citizens who are homeless and unable to feed themselves without assistance and group 2 comprising citizens living in rented accommodation without fixed employment, and can only afford to eat once or twice a day, and group 3 for “middle class” households insluding self-employed citizens small or medium-scale businesses [37,38].

4 *n* = 277 without missing data (*n* = 4).

5 Higher education (*n* = 7) includes postsecondary education, adult education, literacy school, or parish school.

6 *n* = 280 without missing data (*n* = 1).

### Food group consumption

Tables 2 and 3 present the comparison of DQQ with OWFR and 24hR for the 29 DQQ food groups and 10 MDD-W food groups, respectively. The DQQ showed no difference (*P* > 0.05) in population-consumption–prevalence with OWFR and 24hR for most food groups (20 and 18/29 food groups, respectively) (Table 2). Compared with OWFR and 24hR, the DQQ overestimated (*P* < 0.05) the consumption-prevalence of 6 and 8 of 29 food groups, respectively. Only “nuts and seeds” was overestimated by >10 pp (DQQ compared with OWFR: +22 pp, *P* < 0.05; DQQ compared with 24hR: +33 pp, *P* < 0.05) (Table 2). Compared with OWFR and 24hR, the DQQ underestimated (*P* < 0.05) population-consumption–prevalence of 3 of 29 food groups. Two food groups were underestimated by >10 pp (*P* < 0.05): “white roots, tubers, and plantains” (DQQ compared with OWFR: −11 pp; DQQ compared with 24hR: −10pp), and “other vegetables” (DQQ compared with. OWFR: −16 pp; DQQ compared with 24hR: −13 pp) (Table 2). When “white roots, tubers, and plantains” was aggregated into the corresponding MDD-W food group of “grains, white roots and tubers, or plantains,” the underestimation of population-consumption–prevalence of this aggregated food group by DQQ dropped to –5 pp and –4 pp (*P* < 0.05) against OWFR and 24hR, respectively (Table 3).

**TABLE 2:** Comparison between DQQ-OWFR and DQQ-24hR for collecting population-level food group consumption data among adults in Northern Rwanda (*n* = 281)

Food group		DQQ-OWFR							DQQ-24hR						
		Population-consumption–prevalence (%)			*P*	Agreement			Population-consumption–prevalence (%)			*P*	Agreement		
						Misreporting (%)							Misreporting (%)		
		DQQ	OWFR	Diff		PA	FP	FN	DQQ	24hR	Diff		PA	FP	FN
1	Foods made from grains	50.2	47.7	2.5	0.41	81.1	10.7^2^	8.2	50.2	47.3	2.8	0.31	82.9	10.0	7.1
2	Whole grains	44.1	43.8	0.4	1.00	84.0	8.2	7.8	44.1	41.3	2.8	0.29	84.3	9.3	6.4
3	White roots, tubers, and plantains	81.9	93.2	−11.4^2^	<0.001	87.2	0.7	12.1^2^	81.9	92.2	−10.3^2^	<0.001	86.8	1.4	11.7^2^
4	Legumes	85.4	92.5	−7.1^1^	<0.001	87.9	2.5	9.6	85.4	91.1	−5.7^1^	0.005	89.3	2.5	8.2
5	Vitamin A-rich orange vegetables	20.6	15.7	5.0^1^	0.02	88.6	8.2	3.2	20.6	14.6	6.0^1^	0.002	89.7	8.2	2.1
6	Dark green leafy vegetables	62.3	61.6	0.7	0.87	86.5	7.1	6.4	62.3	54.4	7.8^1^	0.005	80.1	13.9^2^	6.1
7	Other vegetables	52.0	68.3	−16.4^2^	<0.001	71.5	6.0	22.4^2^	52.0	64.8	−12.8^2^	<0.001	69.4	8.9	21.7^2^
8	Vitamin A-rich fruits	7.1	1.8	5.3^1^	<0.001	93.2	6.0	0.7	7.1	3.6	3.6^1^	0.01	95.0	4.3	0.7
9	Citrus	1.8	0.7	1.1	0.25	98.9	1.1	0	1.8	0.4	1.4	0.13	98.6	1.4	0
10	Other fruits	22.4	14.2	8.2^1^	<0.001	86.1	11.0^2^	2.8	22.4	14.6	7.8^1^	<0.001	90.0	8.9	1.1
11	Baked/grain-based sweets	4.3	0.7	3.6^1^	0.002	96.4	3.6	0	4.3	1.4	2.8^1^	0.01	97.2	2.8	0
12	Other sweets	1.4	0.7	0.7	0.69	97.9	1.4	0.7	1.4	0.4	1.1	0.38	98.2	1.4	0.4
13	Eggs	4.3	2.8	1.4	0.22	97.9	1.8	0.4	4.3	2.5	1.8	0.13	97.5	2.1	0.4
14	Cheese	0	0	0	NA	100	0	0	0	0	0	NA	100	0	0
15	Yogurt	3.6	1.8	1.8	0.06	98.2	1.8	0	3.6	2.5	1.1	0.38	98.2	1.4	0.4
16	Processed meats	0	0	0	NA	100	0	0	0	0	0	NA	100	0	0
17	Unprocessed red meat (ruminant)	2.5	1.4	1.1	0.38	98.2	1.4	0.4	2.5	1.8	0.7	0.63	98.6	1.1	0.4
18	Unprocessed red meat (nonruminant)	0	0	0	NA	100	0	0	0	0	0	NA	100	0	0
19	Poultry	1.4	0.4	1.1	0.25	98.9	1.1	0	1.4	0.7	0.7	0.50	99.3	0.7	0
20	Fish and seafood	30.6	34.5	−3.9	0.07	89.0	3.6	7.5	30.6	33.8	−3.2	0.15	89.0	3.9	7.1
21	Nuts and seeds	43.1	20.6	22.4^2^	<0.001	68.3	27.0^2^	4.6	43.1	10.3	32.7^2^	<0.001	63.7	34.5^2^	1.8
22	Packaged ultraprocessed salty snacks	1.8	0	1.8	NA	98.2	1.8	0	1.8	0	1.8	NA	98.2	1.8	0
23	Instant noodles	0	0	0	NA	100	0	0	0	0	0	NA	100	0	0
24	Deep fried foods	3.9	7.1	−3.2	0.09	91.8	2.5	5.7	3.9	3.9	0	1.00	95.7	2.1	2.1
25	Fluid milk	10.0	6.8	3.2^1^	0.02	95.4	3.9	0.7	10.0	6.8	3.2^1^	0.02	95.4	3.9	0.7
26	Sweet tea/coffee/cocoa	6.0	5.0	1.1	0.45	97.5	1.8	0.7	6.0	4.3	1.8	0.18	96.8	2.5	0.7
27	Fruit juice and fruit-flavored drinks	5.0	2.5	2.5	0.07	96.0	3.2	0.7	5.0	1.8	3.2^2^	0.01	96.1	3.6	0.4
28	Sugar-sweetened beverages (soft drinks)	5.0	2.8	2.1	0.11	96.4	2.8	0.7	5.0	3.9	1.1	0.55	96.1	2.5	1.4
29	Fast food	0.7	0	0.7	NA	99.3	0.7	0	0.7	0	0.7	NA	99.3	0.7	0
Total [mean (SD)]		NR	NR	0.9 (6.4)	NA	92.6 (8.4)	4.1 (5.4)	3.3 (5.1)	NR	NR	1.8 (7.4)	NA	92.6 (9.2)	4.6 (6.8)	2.8 (4.8)

Abbreviations: 24hR, multi-pass 24-h open recall excluding food items consumed in amounts <15 g except including <15 g for small dried fish; Diff, percentage point difference in population prevalence of food group consumption between DQQ and 24hR (DQQ−24hR) or DQQ and OWFR (DQQ−OWFR); DQQ, Diet Quality Questionnaire; FN, false negatives; FP, false positives; NA, not applicable; *P*, *P* value of McNemar test; OWFR, observed weighed food records excluding food items consumed in amounts <15 g except for small dried fish; PA, percent agreement coefficient.

1 Proportional difference (McNemar test *P* < 0.05) population-prevalence.

2 Proportional difference *P* < 0.05 and >10 percentage points, or overreporting (FP) or underreporting (FN) >10%.

**TABLE 3:** Comparison between DQQ-OWFR and DQQ-24hR for collecting population-level MDD-W food group consumption data among adults in Northern Rwanda (*n* = 281)

MDD-W food group		DQQ-OWFR							DQQ-24hR						
		Population-consumption–prevalence (%)			*P*	Agreement			Population-consumption–prevalence (%)			*P*	Agreement		
						Misreporting (%)							Misreporting (%)		
		DQQ	OWFR	Diff		PA	FP	FN	DQQ	24hR	Diff		PA	FP	FN
1	Grains, white roots and tubers, and plantains	95.0	99.6	−4.6^1^	<0.001	95.4	0	4.6	95.0	99.3	−4.3^1^	0.001	95.0	0.4	4.6
2	Pulses (beans, peas, and lentils)	85.4	92.5	−7.1^1^	<0.001	87.9	2.5	9.6	85.4	91.1	−5.7^1^	0.005	89.3	2.5	8.2
3	Nuts and seeds	43.1	20.6	22.4^2^	<0.001	68.3	27.0^2^	4.6	43.1	10.3	32.7^2^	<0.001	63.7	34.5^2^	1.8
4	Dairy	12.1	8.5	3.6^1^	0.01	95.0	4.3	0.7	12.1	8.9	3.2^1^	0.02	95.4	3.9	0.7
5	Meat, poultry, and fish	32.4	35.6	−3.2	0.16	88.3	4.3	7.5	32.4	35.2	−2.8	0.22	88.6	4.3	7.1
6	Eggs	4.3	2.8	1.4	0.22	97.9	1.8	0.4	4.3	2.5	1.8	0.13	97.5	2.1	0.4
7	Dark green leafy vegetables	62.3	61.6	0.7	0.87	86.5	7.1	6.4	62.3	54.4	7.8^1^	0.005	80.1	13.9^2^	6.1
8	Other vitamin A-rich fruits and vegetables	26.3	16.7	9.6^1^	<0.001	83.3	13.2^2^	3.6	26.3	17.4	8.9^1^	<0.001	85.4	11.7^2^	2.8
9	Other vegetables	52.0	68.3	−16.4^2^	<0.001	71.5	6.0	22.4^2^	52.0	64.8	−12.8^2^	<0.001	69.4	8.9	21.7^2^
10	Other fruits	23.8	14.9	8.9^1^	<0.001	85.4	11.7	2.8	23.8	14.9	8.9^1^	<0.001	89.0	10.0	1.1
Total, mean (SD)		NR	NR	1.5 (10.6)	NA	86.0 (9.7)	7.8 (7.9)	6.3 (6.4)	NR	NR	3.8 (12.4)	NA	85.3 (11.2)	9.2 (10.0)	5.5 (6.3)

Abbreviations: 24hR, multi-pass 24-h open recall excluding food items consumed in amounts <15 g except including <15 g for small dried fish; Diff, percentage point difference in population prevalence of food group consumption between DQQ and 24hR (DQQ−24hR) or DQQ and OWFR (DQQ−OWFR); DQQ, diet quality questionnaire; FN, false negatives; FP, false positives; MDD-W, Minimum Dietary Diversity for Women; NA, not applicable; NR, not relevant; OWFR, observed weighed food records excluding food items consumed in amounts <15 g except for small dried fish; PA, percent agreement coefficient.

1 Proportional difference (McNemar test *P* < 0.05) population-prevalence; *P*, *P* value of McNemar test.

2 Proportional difference *P* < 0.05 and >10 percentage points, or overreporting (FP) or underreporting (FN) >10%.

Mean PA for consumption data of all 29 food groups was 93% between DQQ-OWFR and between DQQ-24hR (Table 2). When combining DQQ food groups into the MDD-W food groups, mean PA for consumption data of the MDD-W food groups was 86% between DQQ-OWFR and 85% between DQQ-24hR (Table 3). The FP rate exceeded 10% for 3 of 29 food groups between DQQ-OWFR: “foods made from grains” (11%), “other fruits” (11%), and “nuts and seeds” (27%) (Table 2). When aggregated into the MDD-W food groups, the FP rate between DQQ-OWFR of “grains, white roots and tubers, and plantains” reduced to 0%, the FP rate of “other fruits” was 12%, and that of “nuts and seeds” was the same (27%), also for DQQ-24hR (35%) (Table 3). In the aggregated MDD-W food group “other vitamin A-rich fruits and vegetables,” the FP rate also exceeded between DQQ-OWFR (13%) and between DQQ-24hR (12%). The FN rate exceeded 10% for 2 of 29 food groups between DQQ-OWFR and DQQ-24hR: “white roots and tubers” (12%), and “other vegetables” (22%) (Table 2). When combined into the MDD-W food groups, the FN rate between DQQ-OWFR and DQQ-24hR of “grains, white roots and tubers, and plantains” reduced to 5% (Table 3). The FN rate between DQQ-OWFR and DQQ-24hR of “other vegetables” stayed the same because the food group is not further aggregated in MDD-W (Table 3).

### Diet quality indicators

For MDD-W, the difference in population-prevalence of females achieving the indicator was 6 pp (*P* > 0.05) between DQQ-OWFR (MDD-W prevalence DQQ: 46%; OWFR: 40%), and +16 pp (*P* < 0.05) between DQQ-24hR (MDD-W prevalence DQQ: 46%; 24hR: 30%) (Table 4). Against OWFR and 24hR, the DQQ overestimated the population-prevalence of adults achieving All-5 (DQQ compared with OWFR: +6 pp, *P* < 0.05; DQQ compared with 24hR: +3 pp, *P* > 0.05), PFC (DQQ compared with OWFR: +9 pp, *P* < 0.05; DQQ compared with 24hR: +8, *P* < 0.05), and underestimated the prevalence of UFC (DQQ compared with OWFR: 4 pp, *P* < 0.05; DQQ compared with 24hR: 3 pp, *P* > 0.05) (Table 4). For continuous indicators, FGDS and NCD-Protect, mean differences between DQQ-OWFR were 0.2 (*P* > 0.05) and 0.2 (*P* = 0.01), respectively (Table 5). Between DQQ-24hR, mean differences in FGDS and NCD-Protect scores were 0.4 (*P* < 0.001) for both indicators. For NCD-Risk, median scores were 0 across OWFR, DQQ, and 24hR.

**TABLE 4:** Comparison between DQQ-OWFR and DQQ-24hR for or achieving binary indicators MDD-W, All-5, PFC, and UFC among adults in Northern Rwanda (*n* = 281)

Indicator	DQQ-OWFR						DQQ-24hR					
	Population-prevalence (%) (95% CI)			Agreement			Population-prevalence (%) (95% CI)			Agreement		
				Misreporting (%)						Misreporting (%)		
	DQQ	OWFR	Diff^1^	PA	FP	FN	DQQ	24hR	Diff^1^	PA	FP	FN
MDD-W^2^	46.0 (38.4, 53.7)	40.4 (33.1, 48.1)	5.6 (−2.3, 13.4)	74.5	15.5^3^	9.9	46.0 (38.4, 53.7)	29.8 (23.3, 37.3)	16.1^3^ (8.0, 23.9)	71.4	22.4^3^	6.2
All-5	12.5 (9.1, 16.9)	6.0 (3.8, 9.5)	6.4^4^ (2.4, 10.3)	88.6	8.9	2.5	12.5 (9.1, 16.9)	9.6 (6.6, 13.7)	2.8 (−0.5, 6.2)	92.2	5.3	2.5
PFC	24.2 (19.5, 29.5)	15.3 (11.5, 20.0)	8.9^4^ (4.0, 13.7)	82.6	13.2^3^	4.3	24.2 (19.5, 29.5)	16.7 (12.8, 21.6)	7.5^4^ (3.1, 11.8)	86.1	10.7^3^	3.2
UFC	7.1 (10.8, 4.6)	3.2 (6.1, 1.6)	3.9^4^ (1.1, 6.7)	94.7	4.6	0.7	7.1 (10.8, 4.6)	4.3 (7.4, 2.4)	2.8 (0.1, 5.7)	94.3	4.3	1.4

Abbreviations: 24hR, multi-pass 24-h open recall excluding food items consumed in amounts <15 g except including <15 g for small dried fish; Diff, percentage point difference in population prevalence of food group consumption between DQQ and 24hR (DQQ−24hR) or between DQQ and OWFR (DQQ−OWFR); CI, confidence interval; DQQ, diet quality questionnaire; FN, false negatives; FP, false positives; MDD-W, minimum dietary diversity for women; OWFR, observed weighed food records excluding food items consumed in amounts <15 g except for small dried fish; PA, percent agreement coefficient; PFC, protective food consumption; UFC, unhealthy food consumption.

1 Values are in percentage point difference in population-prevalence (95% CI).

2 Only calculated for females (*n* = 161).

3 Proportional difference *P* < 0.05 and >10 percentage points, or overreporting (FP) or underreporting (FN) >10%.

4 Proportional difference (McNemar test *P* < 0.05) population-prevalence.

**TABLE 5:** Comparison between DQQ-OWFR and DQQ-24hR of FGDS, NCD-Protect, and NCD-Risk scores among adults in Northern Rwanda (*n* = 281)

Indicator^1^	DQQ-OWFR					DQQ-24hR				
	DQQ^2^	OWFR^2^	Diff in mean	Diff in %	*P*	DQQ^2^	24hR^2^	Diff in mean	Diff in %	*P*
FGDS	4.4 (1.8)	4.2 (1.4)	0.2	1.5	0.059	4.4 (1.8)	4.0 (1.4)	0.4	3.8	<0.001
NCD-Protect	3.4 (1.5)	3.2 (1.2)	0.2	2.2	0.010	3.4 (1.5)	3.0 (1.3)	0.4	4.9	<0.001
NCD-Risk	0 (0–0)	0 (0–0)	—	—	0.045	0 (0–0)	0 (0–0)	—	—	0.002

Abbreviations: 24hR, multi-pass 24-h open recall excluding food items consumed in amounts <15 g except including <15 g for small dried fish; Diff in mean, mean difference in indicator scores between DQQ and 24hR (DQQ minus 24hR), or DQQ and OWFR (DQQ minus OWFR), or 24hR and OWFR (24hR−OWFR); Diff in pp, percentage point difference of indicator scores between DQQ and 24hR (DQQ minus 24hR), or DQQ and OWFR (DQQ−OWFR); DQQ, diet quality questionnaire; FGDS, food group diversity score; NCD, noncommunicable diseases; OWFR, observed weighed food record excluding food items consumed in amounts <15 g except for small dried fish; *P*, *P* value of paired samples *t*-test for FGDS and NCD-Protect, of Wilcoxon signed-rank test for NCD-Risk, and of McNemar’s test for NCD-Risk % consuming >0 food groups of NCD-Risk.

1 Scoring range FGDS: 0–10; NCD-Protect: 0–9; NCD-Risk: 0–9.

2 Values are in mean (SD) for FGDS and NCD-Protect, and in median (25th–75th percentiles) for NCD-Risk.

#### Method of triads to compare the OWFR, DQQ, and 24hR for accurately estimating diet quality indicators

**FIGURE 2:**
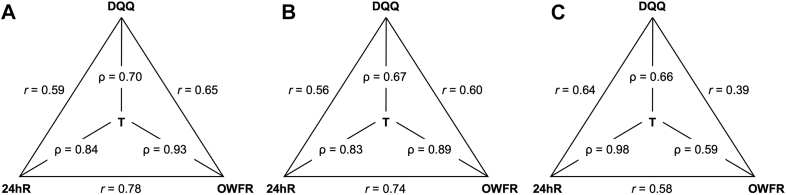
Method of triads between OWFR, DQQ, and 24hR for estimating (A) FGDS (*n* = 281), (B) NCD-Protect (*n* = 281), and (C) NCD-Risk (*n* = 281). 24hR, multi-pass 24-h open recall excluding food items consumed in amounts <15 g except including <15 g for small dried fish; DQQ, Diet Quality Questionnaire; FGDS, Food Group Diversity Score; OWFR, observed weighed food records excluding food items consumed in amounts <15 g except for small dried fish; r, Pearson correlation coefficient between the diet quality indicator score calculated between DQQ / 24hR/ OWFR; T, true but unknown diet quality indicator score; ρ, validity coefficient of diet quality indicator score calculated from DQQ / 24hR/ OWFR in relation to T.

Validity coefficients for accurately estimating diet quality indicators of FGDS, NCD-Protect, and NCD-Risk of OWFR were 0.93 (high), 0.89 (high), and 0.59 (moderate), respectively, of DQQ were 0.70 (moderate), 0.67 (moderate), and 0.66 (moderate), respectively, and of 24hR were 0.84 (high), 0.83 (high), and 0.98 (high), respectively (Figure 2A–C, or Supplemental Table 12).

Overall, compared with the total study population, separate analyses of adult females and males mostly showed comparable results in terms of differences in indicator scores, differences in population-level food group consumption, PA, and misreporting (FP and FN rates) of DQQ-OWFR and DQQ-24hR, as well as outcomes in methods of triads (Supplemental Tables 7–11).

## Discussion

We aimed to evaluate criterion validity of the DQQ in an LMIC context for collecting population-level food group consumption data and estimating diet quality among adults compared with OWFR and 24hR. This is the first evaluation study to collect data using OWFR, DQQ, and 24hR for the same day among adults, which allowed for applying the method of triads to investigate validity of the DQQ to accurately estimate diet quality. Although previous validation studies of the DQQ have used 24hR as a reference method [3,7], this is the first study comparing the DQQ with observed intakes (OOWFR), closer to a reference standard [1,2]. For most food groups, we saw no differences (*P* > 0.05) in population-level food group consumption prevalences. For diet quality prevalence indicators, the DQQ overestimated prevalence by 4 to 9 pp. The DQQ also showed high agreement (93%) with OWFR and 24hR for collecting population-level food group consumption data. On the basis of the method of triads the DQQ also showed moderate performance for accurately estimating continuous diet quality scores in this study population. Overall, the results we present are conservative because we have not adjusted for multiple comparisons for the various indicators used.

Having used both OWFR and 24hR for evaluating DQQ allowed us to investigate whether respondents might have correctly reported consumption of certain food groups in the DQQ but might have forgotten to recall them when not specifically probed for during 24hR. Although previous research showed that 24hR tends to underestimate episodically consumed foods such as fruits, snacks, and beverages compared with OWFR [[8], [9], [10]], this was not observed in our study. In previous studies in other LMICs, the DQQ was found to overestimate (>10 pp) consumption-prevalence of fruits (“citrus”), vegetables (“vitamin A-rich orange vegetables”), tubers (“white roots, tubers, and plantains”), and sweets (“baked/grain-based sweets,” and “other sweets”) when compared with 24hR, but diet quality indicators (including MDD-W) were overestimated to a lesser degree (<10 pp) by DQQ [3,7]. In our current study, we did not observe large overestimations by the DQQ in any of these food groups compared with either OWFR or 24hR, though similar to Uyar et al. [3], diet quality indicators in this study were also slightly overestimated by DQQ compared with OWFR. The DQQ estimated population-prevalence of females achieving MDD-W more accurately than 24hR, compared with OWFR. This was due to a common limitation of 24hR of underestimating portion sizes [9,39,40], which resulted in certain foods (e.g. dark green leafy vegetables) not being counted in MDD-W calculation (when reported 24hR consumption was <15 g, Supplemental Table 13). Thus, this indicates that when criterion validity of DQQ is assessed compared with 24hR as the reference method among adults, it is likely that the extent of “overestimation” of diet quality indicators at population level by DQQ is smaller than observed. Hanley-Cook et al. [28,41] found list-based questionnaires overestimating MDD-W prevalence by a large extent (>10 pp) against OWFR partly due to affirmatively answering food group questions while having consumed <15 g for items of e.g. “nuts and seeds,” “legumes,” and “other vegetables.” In our current study, we also observed large overestimations in population-consumption–prevalence by DQQ of “nuts and seeds” compared with OWFR and 24hR, but not for other food groups. For “nuts and seeds,” the reason for overestimation in population-consumption–prevalence was explained by dried groundnut powder: 72% of “nuts and seeds” consumers consumed it in <15 g mainly in the form of groundnut flour as an ingredient in stews. Respondents may have been particularly aware of ingredients consumed in this study, due to having paid close attention to each ingredient during the OWFR process.

We observed underestimation of population-consumption–prevalence of “other vegetables” by DQQ in this study, compared with both OWFR and 24hR. This was explained by a sentinel food item (chayote) not appearing in the DQQ question for Rwanda, which resulted in systematic underreporting (FN rate >10 pp) of the food group. Missing sentinel food items also occurred in previous subnational validation studies of DQQ regarding food groups with a high variety of food items (particularly vegetables) [3,7]. In our study population, the item chayote was not covered in the DQQ question, but was consumed based on OWFR and 24hR by ∼80% of underreporters. Although this seemed not to have impacted diet quality indicators meaningfully, we recommend adding chayote to the question of “other vegetables” of Rwanda’s DQQ based on consumption in this population. It is important to note that the DQQ was designed for national use, while this study was conducted in a subpopulation in 1 local area of the country. When the DQQ is used at subnational level, especially in small local areas, we recommended reviewing the DQQ items (particularly vegetables) and if any local items are missing, adding them to the questionnaire at the end or at the end of a subsection (e.g. right after the vegetables questions in case missing items belong to vegetables).

As discussed in the methods section, we allowed the inclusion of dried small fish even when consumed in amounts <15 g, because of its critical role in micronutrient adequacy in this population, providing 100% of B12 even in amounts of 7 g. In other contexts, it may also be important to distinguish between dried items that are important sources of micronutrients even when consumed in small amounts (<15 g), and those that are consumed in small amounts for adding flavor, to achieve the purpose of the MDD-W indicator as a proxy for micronutrient adequacy [23,42]. Further research should investigate whether including such small but nutrient-dense items actually improves correlations between food group diversity scores and mean probability of adequacy of micronutrients, to confirm the contextual inclusion of these items in DQQ. Without careful analysis of their nutrient contributions, there is a risk that the DQQ could overestimate MDD-W prevalence in countries where small items are included in the questionnaire.

A tendency toward overestimating food group consumption prevalences in the DQQ resulted in higher prevalence indicators, on the order of 4 to 9 pp. Overreporting consumption of food groups could be due to several potential explanations. First, affirmatively answering DQQ-questions while having consumed small amounts (<15 g) of the sentinel food items as was found in “nuts and seeds” resulted in overestimating consumption prevalences (as discussed earlier). Second, telescoping bias, i.e. reporting consumption of foods while in reality not having consumed them during the DQQ’s recall period (“yesterday”) but during different days [43]. Third, reconstruction bias, as respondents were not systematically guided in DQQ to recall specific aspects of the recall period (e.g. meals (breakfast/lunch/dinner), time and location of consumption) which could have contributed to both over- and underestimating consumption prevalences [44,45], unlike the multiple-pass 24hR, which prompts detailed reconstruction of the recall day [2,21]. Lastly, a capacity overload of working memory, triggered when adults are asked to recall >4 items per question [46,47], which may have contributed both over- and underestimating consumption prevalences for DQQ-questions containing >4 sentinel foods (e.g. “white roots, tubers, and plantains” with 6 items in the Rwandan DQQ-question was underestimated in this study compared with both OWFR and 24hR, but overestimated by DQQ among females in Solomon Islands (7 items in DQQ-question [48]) compared with 24hR [3]). Further research is needed to investigate whether splitting the DQQ into shorter questions with maximum 4 sentinel foods per question improves accuracy of recalling food group consumption. Any updates, however, would have to be done without compromising the tool’s simple and rapid characteristics for dietary assessment at population level.

A strength of this study is that the DQQ was not only compared with the OWFR but also to 24hR for collecting population-level food group consumption data and estimating diet quality. This design enabled us to *1*) investigate whether food groups that are common for 24hR to underestimate, were accurately recalled by DQQ, by comparison to OWFR (as earlier discussed), and *2*) to apply the method of triads to give us an insight into performance of OWFR, DQQ, and 24hR for accurately estimating diet quality indicators. The method of triads shows that DQQ performs moderately in accurately estimating the indicators, whereas OWFR and 24hR perform better. However, we likely overestimated the validity coefficients due to violation of the assumption of independence in random errors between OWFR, DQQ, and 24hR. The DQQ and 24hR both rely on memory to recall dietary intake of the day before and could be affected by social desirability and approval bias [8] and are thereby subject to similar measurement errors [2,4]. In addition, 24hR and OWFR also share potential sources of error, e.g. both may be biased in the same direction and to a similar degree by respondents’ lack of motivation to recall precisely all foods actually consumed in 24hR, and by altering their cooking and/or food intake to simplify the OWFR procedure and/or due to an enumerator being in the house from morning till evening known as “reactivity bias” [39]. All these correlated random errors could have led to overall overestimating the validity coefficients of OWFR, DQQ, and 24hR for accurately estimating diet quality. We therefore also calculated validity coefficients of OWFR, DQQ, and 24hR for estimating FGDS, NCD-Protect, and NCD-Risk using lowest bounds of the 95% confidence interval (CI) of the correlation coefficients, though validity coefficients were similar (Supplemental Table 12). Limitations of the method of triads (related to correlated random errors) include the occurance of negative correlations, which would not allow for calculating validity coefficients, and Heywood cases (validity coefficients >1) [12]. Nonetheless, we did not observe these instances in our analyses.

Another limitation of this study is due to the low-income setting. Consistent with our previous study in LMICs [3], NCD-Risk food groups such as red meat, sweets, packaged ultraprocessed salty snacks, soft drinks, and fast food were consumed by either no one or only small proportions of the study population, which makes it difficult to assess validity of the DQQ to collect consumption data for these food groups (and consequently estimate NCD-Risk) as compared with contexts where they might be consumed by more people such as in high-income countries [49]. Other validation studies in high-income settings could show additional findings regarding these food groups. Exploring criterion validity of the DQQ among other populations such as school-aged children and adolescents (who might recall their diets differently [50]), remains to be further investigated.

In conclusion, the DQQ tends to slightly overestimate diet quality indicator scores against OWFR. Population-prevalence of females achieving MDD-W was better captured by DQQ than 24hR, compared with OWFR, mainly due to underestimating quantities consumed in 24hR (e.g. dark green leafy vegetables). The DQQ showed moderate performance for accurately estimating diet quality indicators with moderate validity coefficients from the method of triads. Our findings show that DQQ is a valid tool for collecting population-level food group consumption data and estimating diet quality.

## Author contributions

The authors’ responsibilities were as follows – BTMU, IDB, AWH, RM, EJMF, EFT: designed research; RM, MGD, RH, KJB-vdB: conducted research; RM, MGD, RH, KJB-vdB: provided essential materials; BTMU: analyzed data and performed statistical analysis; BTMU, IDB, AWH, EFT: wrote paper; BTMU, IDB, AWH, EFT: had primary responsibility for final content; and all authors: read and approved the final manuscript.

## Funding

The research is jointly funded by The Rockefeller Foundation (grant number: 2023-FOD-004) and the CGIAR (grant number: Research Initiative on Digital Innovation) with grants to the International Institute of Tropical Agriculture; and the European Union (EU) and BMZ via GIZ (grant number: 81249667) with a grant to Wageningen University & Research, Agrotechnology & Food Sciences Group, Division of Human Nutrition and Health.

## Conflict of interest

The authors report no conflicts of interest.
