# Supplementary material for: Validity of the Diet Quality Questionnaire Compared with Observed Intake for Estimating Population-Level Diet Quality in Rwandan Adults
Source: Curr Dev Nutr. 2025 Dec 26;10(2):107628. doi: 10.1016/j.cdnut.2025.107628 (PMC12887389; doi:10.1016/j.cdnut.2025.107628)
Supplement: Multimedia component 1 [file mmc1.docx]

**Supplementary material**

**Validity of the Diet Quality Questionnaire versus observed intake for estimating population-level diet quality in Rwandan adults**

Betül T. M. Uyar, et al.


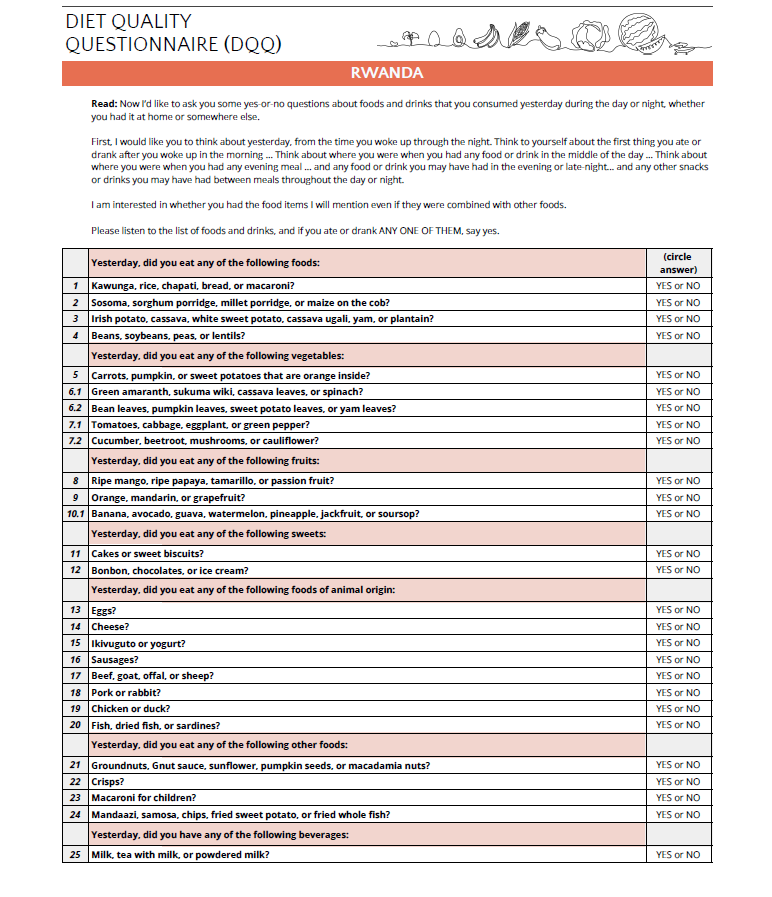
**Supplementary material**


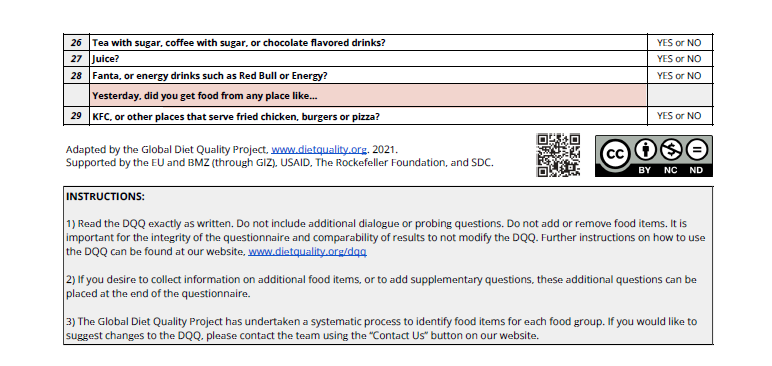


**Supplementary material**

**Table 1.** Median (25^th^ – 75^th^ percentiles) food group consumption (g) and total energy (kcal) intake from 24hR and OWFR of adults in Northern Rwanda (n=281).

| **DQQ food group** | | **24hR** | **OWFR** |
| --- | --- | --- | --- |
| 1 | Foods made from grains | 165 (109 - 250) | 174 (117 – 247) |
| 2 | Whole grains | 105 (54 – 108) | 83 (58 – 168) |
| 3 | White roots, tubers, and plantains | 607 (422 – 954) | 766 (533 – 1096) |
| 4 | Legumes | 211 (130 – 330) | 274 (169 – 426) |
| 5 | Vitamin A-rich orange vegetables | 88 (29 – 454) | 105 (46 – 283) |
| 6 | Dark green leafy vegetables | 49 (25 – 102) | 81 (54 – 149) |
| 7 | Other vegetables | 169 (48 – 327) | 157 (58 – 381) |
| 8 | Vitamin A-rich fruits | 405 (89 – 432) | 62 (34 – 357) |
| 9 | Citrus | 49^a^ | 84 (70 – 98) |
| 10 | Other fruits | 197 (138 – 263) | 122 (87 – 238) |
| 11 | Baked / grain-based sweets | 56 (54 – 63) | 139^a^ |
| 12 | Other sweets | 30^a^ | 245^a^ |
| 13 | Eggs | 53 (46 – 61) | 32 (14 – 64) |
| 14 | Cheese | - | - |
| 15 | Yogurt | 594 (537 – 779) | 594 (573 – 844) |
| 16 | Processed meats | - | - |
| 17 | Unprocessed red meat (ruminant) | 90 (80 – 170) | 240 (228 – 251) |
| 18 | Unprocessed red meat (non-ruminant) | - | - |
| 19 | Poultry | 84 (84 – 84) | 83^a^ |
| 20 | Fish and seafood | 10 (6 – 17) | 11 (6 – 23) |
| 21 | Nuts and seeds | 11 (5 – 17) | 16 (9 – 24) |
| 22 | Packaged ultra-processed salty snacks | - | - |
| 23 | Instant noodles | - | - |
| 24 | Deep fried foods | 152 (119 – 427) | 417 (263 – 620) |
| 25 | Fluid milk | 414 (252 – 583) | 328 (176 – 502) |
| 26 | Sugar-sweetened beverages (soft drinks) | 398 (316 – 646) | 425 (319 – 873) |
| 27 | Fruit juice and fruit-flavored drinks | 999 (625 – 1000) | 826 (565 – 1672) |
| 28 | Sweet tea / coffee / cocoa | 500 (340 – 546) | 324 (239 – 403) |
| 29 | Fast food | - | - |
| **Energy (kcal)** | | 1863  (1361 – 2448) | 2068  (1505 – 2600) |

24hR, 24-hour open recall; DQQ: Diet Quality Questionnaire; OWFR, observed weighed food records. ^a^ Consumption (g) of n=1.

**Supplementary material**

**Table 2a.** Comparison between DQQ-OWFR, DQQ-24hR, and 24hR-OWFR for collecting population-prevalence of consumption of “fish and seafood” and “meat, poultry, and fish” food groups, and reaching MDD-W and All-5 **excluding food items consumed in <15g** among adult women and men in Northern Rwanda.

| **Food group or indicator (%)** | **Population^1^** | **DQQ-24hR** | | | | | | **DQQ-OWFR** | | | | | | **24hR-OWFR** | | | | | |
| --- | --- | --- | --- | --- | --- | --- | --- | --- | --- | --- | --- | --- | --- | --- | --- | --- | --- | --- | --- |
|  |  | Population-  prevalence (%) | | | Agreement | | | Population-  prevalence (%) | | | Agreement | | | Population-  prevalence (%) | | | Agreement | | |
|  |  |  |  |  |  | Misreporting (%) | |  |  |  |  | Misreporting (%) | |  |  |  |  | Misreporting (%) | |
|  |  | DQQ | OWFR | Diff | PA | FP | FN | DQQ | 24hR | Diff | PA | FP | FN | 24hR | OWFR | Diff | PA | FP | FN |
| Fish and seafood  *(food group)* | Women | 29.8 | 9.3 | 20.5** | 78.3 | 21.1** | 0.6 | 29.8 | 6.8 | 23.0** | 73.3 | 24.8** | 1.9 | 6.8 | 9.3 | -2.5 | 87.6 | 5.0 | 7.5 |
|  | Men | 31.7 | 15.8 | 15.8** | 79.2 | 18.3** | 2.5 | 31.7 | 6.7 | 25.0** | 71.7 | 26.7** | 1.7 | 6.7 | 15.8 | -9.2* | 87.5 | 1.7 | 10.8** |
|  | Total | 30.6 | 12.1 | 18.5** | 78.6 | 19.9** | 1.4 | 30.6 | 6.8 | 23.8** | 72.6 | 25.6** | 1.8 | 6.8 | 12.1 | -5.3* | 87.5 | 3.6 | 8.9 |
| Meat, poultry, and fish  *(food group)* | Women | 31.7 | 11.2 | 20.5** | 77.0 | 21.7** | 1.2 | 31.7 | 8.7 | 23.0** | 72.0 | 25.5** | 2.5 | 8.7 | 11.2 | -2.5 | 87.6 | 5.0 | 7.5 |
|  | Men | 33.3 | 16.7 | 16.7** | 78.3 | 19.2** | 2.5 | 33.3 | 9.2 | 24.2** | 72.5 | 25.8** | 1.7 | 9.2 | 16.7 | -7.5* | 85.8 | 3.3 | 10.8** |
|  | Total | 32.4 | 13.5 | 18.9** | 77.6 | 20.6** | 1.8 | 32.4 | 8.9 | 23.5** | 72.2 | 25.6** | 2.1 | 8.9 | 13.5 | -4.6* | 86.8 | 4.3 | 8.9 |
| MDD-W  *(indicator)* | Women | 46.0 | 29.8 | 16.1** | 70.2 | 23.0** | 6.8 | 46.0 | 21.7 | 24.2** | 65.8 | 29.2** | 5.0 | 21.7 | 29.8 | -8.1* | 84.5 | 3.7 | 11.8** |
|  | Men | 45.0 | 32.5 | 12.5** | 74.2 | 19.2** | 6.7 | 45.0 | 28.3 | 16.7** | 73.3 | 21.7** | 5.0 | 28.3 | 32.5 | -4.2 | 84.2 | 5.8 | 10.0 |
|  | Total | 45.6 | 39.5 | 6.0 | 75.4 | 15.3** | 9.3 | 45.6 | 32.0 | 13.5** | 73.7 | 19.9** | 6.4 | 32.0 | 39.5 | -7.5* | 86.8 | 2.8 | 10.3** |
| All-5  *(indicator)* | Women | 11.2 | 2.5 | 8.7* | 88.8 | 9.9 | 1.2 | 11.2 | 2.5 | 8.7* | 90.1 | 9.3 | 0.6 | 2.5 | 2.5 | 0 | 96.3 | 1.9 | 1.9 |
|  | Men | 14.2 | 7.5 | 6.7 | 85.0 | 10.8** | 4.2 | 14.2 | 5.0 | 9.2* | 87.5 | 10.8** | 1.7 | 5.0 | 7.5 | -2.5 | 94.2 | 1.7 | 4.2 |
|  | Total | 12.5 | 4.6 | 7.8* | 87.2 | 10.3** | 2.5 | 12.5 | 3.6 | 8.9* | 89.0 | 10.0 | 1.1 | 3.6 | 4.6 | -1.1 | 95.4 | 1.8 | 2.8 |

24hR, multi-pass 24-hour open recall excluding food items consumed in <15g; Diff, Percentage point difference in population prevalence of food group consumption between DQQ and 24hR (DQQ minus 24hR), between DQQ and OWFR (DQQ minus OWFR), or between 24hR and OWFR (24hR minus OWFR); DQQ, Diet Quality Questionnaire; FN, false negatives; FP, false positives; MDD-W, Minimum Dietary Diversity for Women; PA, percent agreement coefficient; OWFR, observed weighed food records excluding food items consumed in <15g.

^1^ Women n=161; men n=120; total n=281.

* Proportional difference (McNemar test p<0.05) between tools in population-prevalence of food group or indicator. ** Proportional difference p<0.05 and >10 percentage points, or overreporting (FP) or underreporting (FN) >10%.

**Supplementary material**

**Table 2b.** Comparison between DQQ-OWFR, DQQ-24hR, and 24hR-OWFR for estimating FGDS **excluding food items consumed in <15g** among adult women and men in Northern Rwanda.

|  | **DQQ-OWFR** | | | | | **DQQ-24hR** | | | | | **24hR-OWFR** | | | | |
| --- | --- | --- | --- | --- | --- | --- | --- | --- | --- | --- | --- | --- | --- | --- | --- |
| **Population** | DQQ^1^ | OWFR^1^ | Diff  in mean | Diff  in % | p | DQQ^1^ | 24hR^1^ | Diff  in mean | Diff  in % | p | 24hR^1^ | OWFR^1^ | Diff  in mean | Diff  in % | p |
| Women (n=161) | 4.3 (1.8) | 4.0 (1.1) | 0.4 | 3.7 | 0.002 | 4.3 (1.8) | 3.7 (1.2) | 0.6 | 6.4 | <0.001 | 3.7 (1.2) | 4.0 (1.1) | -0.3 | -2.7 | 0.001 |
| Men (n=120) | 4.4 (1.8) | 4.1 (1.4) | 0.4 | 3.8 | 0.003 | 4.4 (1.8) | 3.8 (1.4) | 0.6 | 6.4 | <0.001 | 3.8 (1.4) | 4.1 (1.4) | -0.3 | -2.6 | 0.003 |
| Total (n=281) | 4.4 (1.8) | 4.0 (1.3) | 0.4 | 3.7 | <0.001 | 4.4 (1.8) | 3.7 (1.3) | 0.6 | 6.4 | <0.001 | 3.7 (1.3) | 4.0 (1.3) | -0.3 | -2.7 | <0.001 |

24hR, multi-pass 24-hour open recall excluding food items consumed in amounts <15g; Diff in mean, mean difference in indicator scores between DQQ and 24hR (DQQ minus 24hR), or DQQ and OWFR (DQQ minus OWFR), or 24hR and OWFR (24hR minus OWFR); Diff in pp, Percentage point difference of indicator scores between DQQ and 24hR (DQQ minus 24hR), or DQQ and OWFR (DQQ minus OWFR), or 24hR and OWFR (24hR minus OWFR); DQQ, Diet Quality Questionnaire; FGDS, Food Group Diversity Score (scoring range: 0 – 10); p, p-value of paired samples t-test; OWFR, observed weighed food record excluding food items consumed in amounts <15g.

^1^Values are in mean (standard deviation).

**Supplementary material**

**Table 3.** Comparison between 24hR-OWFR for collecting population-level food group consumption data among adults in Northern Rwanda (n=281).

| **Food group** | | Population-consumption- prevalence (%) | | | Agreement | | |
| --- | --- | --- | --- | --- | --- | --- | --- |
|  |  |  |  |  |  | Misreporting (%) | |
|  |  | 24hR | OWFR | Diff | PA | FP | FN |
| 1 | Foods made from grains | 47.3 | 47.7 | -0.4 | 96.1 | 1.8 | 2.1 |
| 2 | Whole grains | 41.3 | 43.8 | -2.5 | 90.4 | 3.6 | 6.0 |
| 3 | White roots, tubers, and plantains | 92.2 | 93.2 | -1.1 | 97.5 | 0.7 | 1.8 |
| 4 | Legumes | 91.1 | 92.5 | -1.4 | 95.7 | 1.4 | 2.8 |
| 5 | Vitamin A-rich orange vegetables | 14.6 | 15.7 | -1.1 | 96.1 | 1.4 | 2.5 |
| 6 | Dark green leafy vegetables | 54.4 | 61.6 | -7.1* | 85.8 | 3.6 | 10.7** |
| 7 | Other vegetables | 64.8 | 68.3 | -3.6* | 92.9 | 1.8 | 5.3 |
| 8 | Vitamin A-rich fruits | 3.6 | 1.8 | 1.8 | 97.5 | 2.1 | 0.4 |
| 9 | Citrus | 0.4 | 0.7 | -0.4 | 99.6 | 0 | 0.4 |
| 10 | Other fruits | 14.6 | 14.2 | 0.4 | 91.8 | 4.3 | 3.9 |
| 11 | Baked/grain-based sweets | 1.4 | 0.7 | 0.7 | 99.3 | 0.7 | 0 |
| 12 | Other sweets | 0.4 | 0.7 | -0.4 | 99.6 | 0 | 0.4 |
| 13 | Eggs | 2.5 | 2.8 | -0.4 | 99.6 | 0 | 0.4 |
| 14 | Cheese | 0 | 0 | 0 | 100 | 0 | 0 |
| 15 | Yogurt | 2.5 | 1.8 | 0.7 | 99.3 | 0.7 | 0 |
| 16 | Processed meats | 0 | 0 | 0 | 100 | 0 | 0 |
| 17 | Unprocessed red meat (ruminant) | 1.8 | 1.4 | 0.4 | 99.6 | 0.4 | 0 |
| 18 | Unprocessed red meat (non-ruminant) | 0 | 0 | 0 | 100 | 0 | 0 |
| 19 | Poultry | 0.7 | 0.4 | 0.4 | 99.6 | 0.4 | 0 |
| 20 | Fish and seafood | 33.8 | 34.5 | -0.7 | 98.6 | 0.4 | 1.1 |
| 21 | Nuts and seeds | 10.3 | 20.6 | -10.3** | 84.0 | 2.8 | 13.2** |
| 22 | Packaged ultra-processed salty snacks | 0 | 0 | 0 | 100 | 0 | 0 |
| 23 | Instant noodles | 0 | 0 | 0 | 100 | 0 | 0 |
| 24 | Deep fried foods | 3.9 | 7.1 | -3.2* | 93.2 | 1.8 | 5.0 |
| 25 | Fluid milk | 6.8 | 6.8 | 0 | 97.9 | 1.1 | 1.1 |
| 26 | Sweet tea / coffee / cocoa | 4.3 | 5.0 | -0.7 | 98.6 | 0.4 | 1.1 |
| 27 | Fruit juice and fruit-flavored drinks | 1.8 | 2.5 | -0.7 | 98.6 | 0.4 | 1.1 |
| 28 | Sugar-sweetened beverages (soft drinks) | 3.9 | 2.8 | 1.1 | 98.2 | 1.4 | 0.4 |
| 29 | Fast food | 0 | 0 | 0 | 100 | 0 | 0 |
| *Total (mean (SD))* | | *NR* | *NR* | *-1.0 (2.5)* | *96.9 (4.3)* | *1.1 (1.2)* | *2.1 (3.3)* |

24hR, multi-pass 24-hour open recall excluding food items consumed in amounts <15g except including <15g for small dried fish; Diff, percentage point difference in population prevalence of food group consumption between 24hR and OWFR (24hR minus OWFR); FN, false negatives; FP, false positives; NR, not relevant; PA, percent agreement coefficient; OWFR, observed weighed food records excluding food items consumed in amounts <15g except including <15g for small dried fish.

* Proportional difference (McNemar test p<0.05) population-prevalence.

** Proportional difference p<0.05 and >10 percentage points, or overreporting (FP) or underreporting (FN) >10%.

**Supplementary material**

**Table 4.** Comparison between 24hR-OWFR for collecting population-level MDD-W food group consumption data among adults in Northern Rwanda (n=281).

| **MDD-W food group** | | Population-consumption- prevalence (%) | | | Agreement | | |
| --- | --- | --- | --- | --- | --- | --- | --- |
|  |  |  |  |  |  | Misreporting (%) | |
|  |  | 24hR | OWFR | Diff | PA | FP | FN |
| 1 | Grains, white roots and tubers, and plantains | 99.3 | 99.6 | -0.4 | 99.6 | 0 | 0.4 |
| 2 | Pulses (beans, peas and lentils) | 91.1 | 92.5 | -1.4 | 95.7 | 1.4 | 2.8 |
| 3 | Nuts and seeds | 10.3 | 20.6 | -10.3** | 84.0 | 2.8 | 13.2** |
| 4 | Dairy | 8.9 | 8.5 | 0.4 | 98.2 | 1.1 | 0.7 |
| 5 | Meat, poultry and fish | 35.2 | 35.6 | -0.4 | 98.2 | 0.7 | 1.1 |
| 6 | Eggs | 2.5 | 2.8 | -0.4 | 99.6 | 0 | 0.4 |
| 7 | Dark green leafy vegetables | 54.4 | 61.6 | -7.1* | 85.8 | 3.6 | 10.7** |
| 8 | Other vitamin A-rich fruits and vegetables | 17.4 | 16.7 | 0.7 | 94.3 | 3.2 | 2.5 |
| 9 | Other vegetables | 64.8 | 68.3 | -3.6* | 92.9 | 1.8 | 5.3 |
| 10 | Other fruits | 14.9 | 14.9 | 0 | 91.5 | 4.3 | 4.3 |
| *Total (mean (SD))* | | *NR* | *NR* | *-2.3 (3.7)* | *94.0 (5.5)* | *1.9 (1.5)* | *4.1 (4.5)* |

24hR, multi-pass 24-hour open recall excluding food items consumed in amounts <15g except including <15g for small dried fish; Diff, percentage point difference in population prevalence of food group consumption between 24hR and OWFR (24hR minus OWFR); FN, false negatives; FP, false positives; MDD-W, Minimum Dietary Diversity for Women; NR, not relevant; PA, percent agreement coefficient; OWFR, observed weighed food records excluding food items consumed in amounts <15g except including <15g for small dried fish.

* Proportional difference (McNemar test p<0.05) population-prevalence.

** Proportional difference p<0.05 and >10 percentage points, or overreporting (FP) or underreporting (FN) >10%.

**Supplementary material**

**Table 5.** Comparison between 24hR-OWFR for achieving binary indicators MDD-W, All-5, HDI, PFC, and UFC among adults in Northern Rwanda (n=281).

| **Indicator** | **24hR-OWFR** | | | | | |
| --- | --- | --- | --- | --- | --- | --- |
|  | Population-prevalence (%)  (95% CI) | | | Agreement | | |
|  |  |  |  |  | Misreporting (%) | |
|  | 24hR | OWFR | Diff^1^ | PA | FP | FN |
| MDD-W^2^ | 29.8 (23.3, 37.3) | 40.4 (33.1, 48.1) | -10.6** (-16.7, -4.2) | 83.2 | 3.1 | 13.7** |
| All-5 | 9.6 (6.6, 13.7) | 6.0 (3.8, 9.5) | 3.6* (0.5, 6.6) | 93.6 | 5.0 | 1.4 |
| PFC | 16.7 (12.8, 21.6) | 15.3 (11.5, 20.0) | 1.4 (-2.5, 5.3) | 89.3 | 6.0 | 4.6 |
| UFC | 4.3 (7.4, 2.4) | 3.2 (6.1, 1.6) | 1.1 (-0.8, 2.9) | 98.2 | 1.4 | 0.4 |
| HDI | 15.7 (11.9, 20.4) | 14.6 (10.9, 19.2) | 1.1 (-2.8, 4.9) | 89.7 | 5.7 | 4.6 |

24hR, multi-pass 24-hour open recall excluding food items consumed in amounts <15g except including <15g for small dried fish; Diff, percentage point difference in population prevalence of food group consumption between DQQ and 24hR (DQQ minus 24hR) or between DQQ and OWFR (DQQ minus OWFR); DQQ, Diet Quality Questionnaire; FN, false negatives; FP, false positives; HDI, Healthy Diet Indicator; MDD-W, Minimum Dietary Diversity for Women; PA, percent agreement coefficient; PFC, Protective Food Consumption; UFC, Unhealthy Food Consumption; OWFR, observed weighed food records excluding food items consumed in amounts <15g except including <15g for small dried fish.

* Proportional difference (McNemar test p<0.05) population-prevalence.

** Proportional difference p<0.05 and >10 percentage points, or overreporting (FP) or underreporting (FN) >10%.

^1^ Values are in percentage point difference in population-prevalence (95% CI).

^2^ Only calculated for women (n=161).

**Table 6.** Comparison between 24hR-OWFR of FGDS, NCD-Protect, and NCD-Risk scores among adults in Northern Rwanda (n=281).

| **Indicator^1^** | 24hR^2^ | OWFR^2^ | Diff in mean | Diff in % | p |
| --- | --- | --- | --- | --- | --- |
| FGDS | 4.0 (1.4) | 4.2 (1.4) | -0.2 | -2.2 | <0.001 |
| NCD-Protect | 3.0 (1.3) | 3.2 (1.2) | -0.2 | -2.7 | <0.001 |
| NCD-Risk | 0 (0 – 0) | 0 (0 – 0) | - | - | 0.491 |

24hR, multi-pass 24-hour open recall excluding food items consumed in amounts <15g except including <15g for small dried fish; Diff in mean, mean difference in indicator scores between 24hR and OWFR (24hR minus OWFR); Diff in pp, percentage point difference of indicator scores between 24hR and OWFR (24hR minus OWFR); FGDS, Food Group Diversity Score; NCD, non-communicable diseases; p, p-value of paired samples t-test for FGDS and NCD-Protect, and of Wilcoxon signed-rank test for NCD-Risk; OWFR, observed weighed food record excluding food items consumed in amounts <15g except including <15g for small dried fish.

^1^ Scoring range FGDS: 0 – 10; NCD-Protect: 0 – 9; NCD-Risk: 0 – 9.

^2^ Values are in mean (SD) for FGDS and NCD-Protect, and in median (25^th^ – 75^th^ percentiles) for NCD-Risk.

**Supplementary material**

**Table 7.** Comparison between DQQ-24hR, DQQ-OWFR, and 24hR-OWFR for collecting population-level food group consumption data among adult women and men in Northern Rwanda, **excluding food items consumed in amounts <15g except including <15g for small dried fish in OWFR and 24hR**.

| **Food group** | | **Population^1^** | **DQQ-OWFR** | | | | | | **DQQ-24hR** | | | | | | **24hR-OWFR** | | | | | |
| --- | --- | --- | --- | --- | --- | --- | --- | --- | --- | --- | --- | --- | --- | --- | --- | --- | --- | --- | --- | --- |
|  |  |  | Population-consumption- prevalence (%) | | | Agreement | | | Population-consumption- prevalence (%) | | | Agreement | | | Population-consumption- prevalence (%) | | | Agreement | | |
|  |  |  |  |  |  |  | Misreporting (%) | |  |  |  |  | Misreporting  (%) | |  |  |  |  | Misreporting (%) | |
|  |  |  | DQQ | OWFR | Diff | PA | FP | FN | DQQ | 24hR | Diff | PA | FP | FN | 24hR | OWFR | Diff | PA | FP | FN |
| 1 | Foods made from grains | Women | 53.4 | 54.7 | -1.2 | 80.1 | 9.3 | 10.6** | 53.4 | 53.4 | 0 | 81.4 | 9.3 | 9.3 | 53.4 | 54.7 | -1.2 | 96.3 | 1.2 | 2.5 |
|  |  | Men | 45.8 | 38.3 | 7.5 | 82.5 | 12.5 | 5.0 | 45.8 | 39.2 | 6.7 | 85.0 | 10.8** | 4.2 | 39.2 | 38.3 | 0.8 | 95.8 | 2.5 | 1.7 |
|  |  | Total | 50.2 | 47.7 | 2.5 | 81.1 | 10.7** | 8.2 | 50.2 | 47.3 | 2.8 | 82.9 | 10.0 | 7.1 | 47.3 | 47.7 | -0.4 | 96.1 | 1.8 | 2.1 |
| 2 | Whole grains | Women | 47.8 | 50.3 | -2.5 | 81.4 | 8.1 | 10.6** | 47.8 | 46.0 | 1.9 | 82.0 | 9.9 | 8.1 | 46.0 | 50.3 | -4.3 | 90.7 | 2.5 | 6.8 |
|  |  | Men | 39.2 | 35.0 | 4.2 | 87.5 | 8.3 | 4.2 | 39.2 | 35.0 | 4.2 | 87.5 | 8.3 | 4.2 | 35.0 | 35.0 | 0 | 90.0 | 5.0 | 5.0 |
|  |  | Total | 44.1 | 43.8 | 0.4 | 84.0 | 8.2 | 7.8 | 44.1 | 41.3 | 2.8 | 84.3 | 9.3 | 6.4 | 41.3 | 43.8 | -2.5 | 90.4 | 3.6 | 6.0 |
| 3 | White roots, tubers, and plantains | Women | 80.7 | 91.9 | -11.2** | 86.3 | 1.2 | 12.4** | 80.7 | 90.7 | -9.9* | 85.1 | 2.5 | 12.4** | 90.7 | 91.9 | -1.2 | 96.3 | 1.2 | 2.5 |
|  |  | Men | 83.3 | 95.0 | -11.7** | 88.3 | 0 | 11.7** | 83.3 | 94.2 | -10.8** | 89.2 | 0 | 10.8** | 94.2 | 95.0 | -0.8 | 99.2 | 0 | 0.8 |
|  |  | Total | 81.9 | 93.2 | -11.4** | 87.2 | 0.7 | 12.1** | 81.9 | 92.2 | -10.3** | 86.8 | 1.4 | 11.7** | 92.2 | 93.2 | -1.1 | 97.5 | 0.7 | 1.8 |
| 4 | Legumes | Women | 82.6 | 88.8 | -6.2* | 85.1 | 4.3 | 10.6** | 82.6 | 88.2 | -5.6 | 88.2 | 3.1 | 8.7 | 88.2 | 88.8 | -0.6 | 94.4 | 2.5 | 3.1 |
|  |  | Men | 89.2 | 97.5 | -8.3* | 91.7 | 0 | 8.3 | 89.2 | 95.0 | -5.8 | 90.8 | 1.7 | 7.5 | 95.0 | 97.5 | -2.5 | 97.5 | 0 | 2.5 |
|  |  | Total | 85.4 | 92.5 | -7.1* | 87.9 | 2.5 | 9.6 | 85.4 | 91.1 | -5.7* | 89.3 | 2.5 | 8.2 | 91.1 | 92.5 | -1.4 | 95.7 | 1.4 | 2.8 |
| 5 | Vitamin A-rich orange vegetables | Women | 20.5 | 14.9 | 5.6 | 88.2 | 8.7 | 3.1 | 20.5 | 14.3 | 6.2* | 88.8 | 8.7 | 2.5 | 14.3 | 14.9 | -0.6 | 95.7 | 1.9 | 2.5 |
|  |  | Men | 20.8 | 16.7 | 4.2 | 89.2 | 7.5 | 3.3 | 20.8 | 15.0 | 5.8 | 90.8 | 7.5 | 1.7 | 15.0 | 16.7 | -1.7 | 96.7 | 0.8 | 2.5 |
|  |  | Total | 20.6 | 15.7 | 5.0* | 88.6 | 8.2 | 3.2 | 20.6 | 14.6 | 6.0* | 89.7 | 8.2 | 2.1 | 14.6 | 15.7 | -1.1 | 96.1 | 1.4 | 2.5 |
| 6 | Dark green leafy vegetables | Women | 62.7 | 62.1 | 0.6 | 84.5 | 8.1 | 7.5 | 62.7 | 53.4 | 9.3* | 77.0 | 16.1** | 6.8 | 53.4 | 62.1 | -8.7* | 85.1 | 3.1 | 11.8** |
|  |  | Men | 61.7 | 60.8 | 0.8 | 89.2 | 5.8 | 5.0 | 61.7 | 55.8 | 5.8 | 84.2 | 10.8** | 5.0 | 55.8 | 60.8 | -5.0 | 86.7 | 4.2 | 9.2 |
|  |  | Total | 62.3 | 61.6 | 0.7 | 86.5 | 7.1 | 6.4 | 62.3 | 54.4 | 7.8* | 80.1 | 13.9** | 6.1 | 54.4 | 61.6 | -7.1* | 85.8 | 3.6 | 10.7** |
| 7 | Other vegetables | Women | 56.5 | 73.9 | -17.4** | 71.4 | 5.6 | 23.0** | 56.5 | 70.8 | -14.3** | 70.8 | 7.5 | 21.7** | 70.8 | 73.9 | -3.1 | 94.4 | 1.2 | 4.3 |
|  |  | Men | 45.8 | 60.8 | -15.0** | 71.7 | 6.7 | 21.7** | 45.8 | 56.7 | -10.8** | 67.5 | 10.8** | 21.7** | 56.7 | 60.8 | -4.2 | 90.8 | 2.5 | 6.7 |
|  |  | Total | 52.0 | 68.3 | -16.4** | 71.5 | 6.0 | 22.4** | 52.0 | 64.8 | -12.8** | 69.4 | 8.9 | 21.7** | 64.8 | 68.3 | -3.6* | 92.9 | 1.8 | 5.3 |

24hR, multi-pass 24-hour open recall excluding food items consumed in amounts <15g except including <15g for small dried fish; Diff, percentage point difference in population prevalence of food group consumption between DQQ and 24hR (DQQ minus 24hR) or DQQ and OWFR (DQQ minus OWFR); DQQ, Diet Quality Questionnaire; FN, false negatives; FP, false positives; PA, percent agreement coefficient; OWFR, observed weighed food records excluding food items consumed in amounts <15g except including <15g for small dried fish.

^1^ Women n=161; men n=120; total n=281.

* Proportional difference (McNemar test p<0.05) population-prevalence. ** Proportional difference p<0.05 and >10 percentage points, or overreporting (FP) or underreporting (FN) >10%.

Continued.

**Supplementary material**

**Continued, Table 7**

| **Food group** | | **Population^1^** | **DQQ-OWFR** | | | | | | **DQQ-24hR** | | | | | | **24hR-OWFR** | | | | | |
| --- | --- | --- | --- | --- | --- | --- | --- | --- | --- | --- | --- | --- | --- | --- | --- | --- | --- | --- | --- | --- |
|  |  |  | Population-consumption- prevalence (%) | | | Agreement | | | Population-consumption- prevalence (%) | | | Agreement | | | Population-consumption- prevalence (%) | | | Agreement | | |
|  |  |  |  |  |  |  | Misreporting (%) | |  |  |  |  | Misreporting  (%) | |  |  |  |  | Misreporting (%) | |
|  |  |  | DQQ | OWFR | Diff | PA | FP | FN | DQQ | 24hR | Diff | PA | FP | FN | 24hR | OWFR | Diff | PA | FP | FN |
| 8 | Vitamin A-rich fruits | Women | 6.8 | 1.9 | 5.0* | 93.8 | 5.6 | 0.6 | 6.8 | 2.5 | 4.3* | 94.4 | 5.0 | 0.6 | 2.5 | 1.9 | 0.6 | 99.4 | 0.6 | 0 |
|  |  | Men | 7.5 | 1.7 | 5.8* | 92.5 | 6.7 | 0.8 | 7.5 | 5.0 | 2.5 | 95.8 | 3.3 | 0.8 | 5.0 | 1.7 | 3.3 | 95.0 | 4.2 | 0.8 |
|  |  | Total | 7.1 | 1.8 | 5.3* | 93.2 | 6.0 | 0.7 | 7.1 | 3.6 | 3.6* | 95.0 | 4.3 | 0.7 | 3.6 | 1.8 | 1.8 | 97.5 | 2.1 | 0.4 |
| 9 | Citrus | Women | 2.5 | 1.2 | 1.2 | 98.8 | 1.2 | 0 | 2.5 | 0.6 | 1.9 | 98.1 | 1.9 | 0 | 0.6 | 1.2 | -0.6 | 99.4 | 0 | 0.6 |
|  |  | Men | 0.8 | 0 | 0.8 | 99.2 | 0.8 | 0 | 0.8 | 0 | 0.8 | 99.2 | 0.8 | 0 | 0 | 0 | 0 | 100 | 0 | 0 |
|  |  | Total | 1.8 | 0.7 | 1.1 | 98.9 | 1.1 | 0 | 1.8 | 0.4 | 1.4 | 98.6 | 1.4 | 0 | 0.4 | 0.7 | -0.4 | 99.6 | 0 | 0.4 |
| 10 | Other fruits | Women | 18.6 | 11.2 | 7.5* | 87.6 | 9.9 | 2.5 | 18.6 | 11.2 | 7.5* | 90.1 | 8.7 | 1.2 | 11.2 | 11.2 | 0 | 91.3 | 4.3 | 4.3 |
|  |  | Men | 27.5 | 18.3 | 9.2* | 84.2 | 12.5** | 3.3 | 27.5 | 19.2 | 8.3* | 90.0 | 9.2 | 0.8 | 19.2 | 18.3 | 0.8 | 92.5 | 4.2 | 3.3 |
|  |  | Total | 22.4 | 14.2 | 8.2* | 86.1 | 11.0** | 2.8 | 22.4 | 14.6 | 7.8* | 90.0 | 8.9 | 1.1 | 14.6 | 14.2 | 0.4 | 91.8 | 4.3 | 3.9 |
| 11 | Baked/grain-based sweets | Women | 3.7 | 0 | 3.7 | 96.3 | 3.7 | 0 | 3.7 | 0 | 3.7 | 96.3 | 3.7 | 0 | 0 | 0 | 0 | 100 | 0 | 0 |
|  |  | Men | 5.0 | 1.7 | 3.3 | 96.7 | 3.3 | 0 | 5.0 | 3.3 | 1.7 | 98.3 | 1.7 | 0 | 3.3 | 1.7 | 1.7 | 3.3 | 1.7 | 0 |
|  |  | Total | 4.3 | 0.7 | 3.6* | 96.4 | 3.6 | 0 | 4.3 | 1.4 | 2.8* | 97.2 | 2.8 | 0 | 1.4 | 0.7 | 0.7 | 99.3 | 0.7 | 0 |
| 12 | Other sweets | Women | 1.2 | 1.2 | 0 | 97.5 | 1.2 | 1.2 | 1.2 | 0.6 | 0.6 | 98.1 | 1.2 | 0.6 | 0.6 | 1.2 | -0.6 | 99.4 | 0 | 0.6 |
|  |  | Men | 1.7 | 0 | 1.7 | 98.3 | 1.7 | 0 | 1.7 | 0 | 1.7 | 98.3 | 1.7 | 0 | 0 | 0 | 0 | 100 | 0 | 0 |
|  |  | Total | 1.4 | 0.7 | 0.7 | 97.9 | 1.4 | 0.7 | 1.4 | 0.4 | 1.1 | 98.2 | 1.4 | 0.4 | 0.4 | 0.7 | -0.4 | 99.6 | 0 | 0.4 |
| 13 | Eggs | Women | 3.1 | 2.5 | 0.6 | 99.4 | 0.6 | 0 | 3.1 | 1.9 | 1.2 | 98.8 | 1.2 | 0 | 1.9 | 2.5 | -0.6 | 99.4 | 0 | 0.6 |
|  |  | Men | 5.8 | 3.3 | 2.5 | 95.8 | 3.3 | 0.8 | 5.8 | 3.3 | 2.5 | 95.8 | 3.3 | 0.8 | 3.3 | 3.3 | 0 | 100 | 0 | 0 |
|  |  | Total | 4.3 | 2.8 | 1.4 | 97.9 | 1.8 | 0.4 | 4.3 | 2.5 | 1.8 | 97.5 | 2.1 | 0.4 | 2.5 | 2.8 | -0.4 | 99.6 | 0 | 0.4 |
| 14 | Cheese | Women | 0 | 0 | 0 | 100 | 0 | 0 | 0 | 0 | 0 | 100 | 0 | 0 | 0 | 0 | 0 | 100 | 0 | 0 |
|  |  | Men | 0 | 0 | 0 | 100 | 0 | 0 | 0 | 0 | 0 | 100 | 0 | 0 | 0 | 0 | 0 | 100 | 0 | 0 |
|  |  | Total | 0 | 0 | 0 | 100 | 0 | 0 | 0 | 0 | 0 | 100 | 0 | 0 | 0 | 0 | 0 | 100 | 0 | 0 |

24hR, multi-pass 24-hour open recall excluding food items consumed in amounts <15g except including <15g for small dried fish; Diff, percentage point difference in population prevalence of food group consumption between DQQ and 24hR (DQQ minus 24hR) or DQQ and OWFR (DQQ minus OWFR); DQQ, Diet Quality Questionnaire; FN, false negatives; FP, false positives; PA, percent agreement coefficient; OWFR, observed weighed food records excluding food items consumed in amounts <15g except including <15g for small dried fish.

^1^ Women n=161; men n=120; total n=281.

* Proportional difference (McNemar test p<0.05) population-prevalence. ** Proportional difference p<0.05 and >10 percentage points, or overreporting (FP) or underreporting (FN) >10%.

Continued.

**Supplementary material**

**Continued, Table 7**

| **Food group** | | **Population^1^** | **DQQ-OWFR** | | | | | | **DQQ-24hR** | | | | | | **24hR-OWFR** | | | | | |
| --- | --- | --- | --- | --- | --- | --- | --- | --- | --- | --- | --- | --- | --- | --- | --- | --- | --- | --- | --- | --- |
|  |  |  | Population-consumption- prevalence (%) | | | Agreement | | | Population-consumption- prevalence (%) | | | Agreement | | | Population-consumption- prevalence (%) | | | Agreement | | |
|  |  |  |  |  |  |  | Misreporting (%) | |  |  |  |  | Misreporting  (%) | |  |  |  |  | Misreporting (%) | |
|  |  |  | DQQ | OWFR | Diff | PA | FP | FN | DQQ | 24hR | Diff | PA | FP | FN | 24hR | OWFR | Diff | PA | FP | FN |
| 15 | Yogurt | Women | 1.2 | 0.6 | 0.6 | 99.4 | 0.6 | 0 | 1.2 | 1.2 | 0 | 98.8 | 0.6 | 0.6 | 1.2 | 0.6 | 0.6 | 99.4 | 0.6 | 0 |
|  |  | Men | 6.7 | 3.3 | 3.3 | 96.7 | 3.3 | 0 | 6.7 | 4.2 | 2.5 | 97.5 | 2.5 | 0 | 4.2 | 3.3 | 0.8 | 99.2 | 0.8 | 0 |
|  |  | Total | 3.6 | 1.8 | 1.8 | 98.2 | 1.8 | 0 | 3.6 | 2.5 | 1.1 | 98.2 | 1.4 | 0.4 | 2.5 | 1.8 | 0.7 | 99.3 | 0.7 | 0 |
| 16 | Processed meats | Women | 0 | 0 | 0 | 100 | 0 | 0 | 0 | 0 | 0 | 100 | 0 | 0 | 0 | 0 | 0 | 100 | 0 | 0 |
|  |  | Men | 0 | 0 | 0 | 100 | 0 | 0 | 0 | 0 | 0 | 100 | 0 | 0 | 0 | 0 | 0 | 100 | 0 | 0 |
|  |  | Total | 0 | 0 | 0 | 100 | 0 | 0 | 0 | 0 | 0 | 100 | 0 | 0 | 0 | 0 | 0 | 100 | 0 | 0 |
| 17 | Unprocessed red meat (ruminant) | Women | 1.9 | 1.2 | 0.6 | 98.1 | 1.2 | 0.6 | 1.9 | 1.2 | 0.6 | 98.1 | 1.2 | 0.6 | 1.2 | 1.2 | 0 | 100 | 0 | 0 |
|  |  | Men | 3.3 | 1.7 | 1.7 | 98.3 | 1.7 | 0 | 3.3 | 2.5 | 0.8 | 99.2 | 0.8 | 0 | 2.5 | 1.7 | 0.8 | 99.2 | 0.8 | 0 |
|  |  | Total | 2.5 | 1.4 | 1.1 | 98.2 | 1.4 | 0.4 | 2.5 | 1.8 | 0.7 | 98.6 | 1.1 | 0.4 | 1.8 | 1.4 | 0.4 | 99.6 | 0.4 | 0 |
| 18 | Unprocessed red meat  (non-ruminant) | Women | 0 | 0 | 0 | 100 | 0 | 0 | 0 | 0 | 0 | 100 | 0 | 0 | 0 | 0 | 0 | 100 | 0 | 0 |
|  |  | Men | 0 | 0 | 0 | 100 | 0 | 0 | 0 | 0 | 0 | 100 | 0 | 0 | 0 | 0 | 0 | 100 | 0 | 0 |
|  |  | Total | 0 | 0 | 0 | 100 | 0 | 0 | 0 | 0 | 0 | 100 | 0 | 0 | 0 | 0 | 0 | 100 | 0 | 0 |
| 19 | Poultry | Women | 1.9 | 0.6 | 1.2 | 98.8 | 1.2 | 0 | 1.9 | 0.6 | 1.2 | 98.8 | 1.2 | 0 | 0.6 | 0.6 | 0 | 100 | 0 | 0 |
|  |  | Men | 0.8 | 0 | 0.8 | 99.2 | 0.8 | 0 | 0.8 | 0.8 | 0 | 100 | 0 | 0 | 0.8 | 0 | 0.8 | 99.2 | 0.8 | 0 |
|  |  | Total | 1.4 | 0.4 | 1.1 | 98.9 | 1.1 | 0 | 1.4 | 0.7 | 0.7 | 99.3 | 0.7 | 0 | 0.7 | 0.4 | 0.4 | 99.6 | 0.4 | 0 |
| 20 | Fish and seafood | Women | 29.8 | 37.3 | -7.5* | 88.8 | 1.9 | 9.3 | 29.8 | 35.4 | -5.6 | 88.2 | 3.1 | 8.7 | 35.4 | 37.3 | -1.9 | 98.1 | 0 | 1.9 |
|  |  | Men | 31.7 | 30.8 | 0.8 | 89.2 | 5.8 | 5.0 | 31.7 | 31.7 | 0 | 90.0 | 5.0 | 5.0 | 31.7 | 30.8 | 0.8 | 99.2 | 0.8 | 0 |
|  |  | Total | 30.6 | 34.5 | -3.9 | 89.0 | 3.6 | 7.5 | 30.6 | 33.8 | -3.2 | 89.0 | 3.9 | 7.1 | 33.8 | 34.5 | -0.7 | 98.6 | 0.4 | 1.1 |
| 21 | Nuts and seeds | Women | 45.3 | 20.5 | 24.8** | 66.5 | 29.2** | 4.3 | 45.3 | 9.9 | 35.4** | 62.1 | 36.6** | 1.2 | 9.9 | 20.5 | -10.6** | 80.7 | 4.3 | 14.9** |
|  |  | Men | 40.0 | 20.8 | 19.2** | 70.8 | 24.2** | 5.0 | 40.0 | 10.8 | 29.2** | 65.8 | 31.7** | 2.5 | 10.8 | 20.8 | -10.0* | 88.3 | 0.8 | 10.8** |
|  |  | Total | 43.1 | 20.6 | 22.4** | 68.3 | 27.0** | 4.6 | 43.1 | 10.3 | 32.7** | 63.7 | 34.5** | 1.8 | 10.3 | 20.6 | -10.3** | 84.0 | 2.8 | 13.2** |

24hR, multi-pass 24-hour open recall excluding food items consumed in amounts <15g except including <15g for small dried fish; Diff, percentage point difference in population prevalence of food group consumption between DQQ and 24hR (DQQ minus 24hR) or DQQ and OWFR (DQQ minus OWFR); DQQ, Diet Quality Questionnaire; FN, false negatives; FP, false positives; PA, percent agreement coefficient; OWFR, observed weighed food records excluding food items consumed in amounts <15g except including <15g for small dried fish.

^1^ Women n=161; men n=120; total n=281.

* Proportional difference (McNemar test p<0.05) population-prevalence. ** Proportional difference p<0.05 and >10 percentage points, or overreporting (FP) or underreporting (FN) >10%.

Continued.

**Supplementary material**

**Continued, Table 7**

| **Food group** | | **Population^1^** | **DQQ-OWFR** | | | | | | **DQQ-24hR** | | | | | | **24hR-OWFR** | | | | | |
| --- | --- | --- | --- | --- | --- | --- | --- | --- | --- | --- | --- | --- | --- | --- | --- | --- | --- | --- | --- | --- |
|  |  |  | Population-consumption- prevalence (%) | | | Agreement | | | Population-consumption- prevalence (%) | | | Agreement | | | Population-consumption- prevalence (%) | | | Agreement | | |
|  |  |  |  |  |  |  | Misreporting (%) | |  |  |  |  | Misreporting  (%) | |  |  |  |  | Misreporting (%) | |
|  |  |  | DQQ | OWFR | Diff | PA | FP | FN | DQQ | 24hR | Diff | PA | FP | FN | 24hR | OWFR | Diff | PA | FP | FN |
| 22 | Packaged ultra-processed salty snacks | Women | 1.9 | 0 | 1.9 | 98.1 | 1.9 | 0 | 1.9 | 0 | 1.9 | 98.1 | 1.9 | 0 | 0 | 0 | 0 | 100 | 0 | 0 |
|  |  | Men | 1.7 | 0 | 1.7 | 98.3 | 1.7 | 0 | 1.7 | 0 | 1.7 | 98.3 | 1.7 | 0 | 0 | 0 | 0 | 100 | 0 | 0 |
|  |  | Total | 1.8 | 0 | 1.8 | 98.2 | 1.8 | 0 | 1.8 | 0 | 1.8 | 98.2 | 1.8 | 0 | 0 | 0 | 0 | 100 | 0 | 0 |
| 23 | Instant noodles | Women | 0 | 0 | 0 | 100 | 0 | 0 | 0 | 0 | 0 | 100 | 0 | 0 | 0 | 0 | 0 | 100 | 0 | 0 |
|  |  | Men | 0 | 0 | 0 | 100 | 0 | 0 | 0 | 0 | 0 | 100 | 0 | 0 | 0 | 0 | 0 | 100 | 0 | 0 |
|  |  | Total | 0 | 0 | 0 | 100 | 0 | 0 | 0 | 0 | 0 | 100 | 0 | 0 | 0 | 0 | 0 | 100 | 0 | 0 |
| 24 | Deep fried foods | Women | 3.7 | 7.5 | -3.7 | 91.3 | 2.5 | 6.2 | 3.7 | 4.3 | -0.6 | 95.7 | 1.9 | 2.5 | 4.3 | 7.5 | -3.1 | 94.4 | 1.2 | 4.3 |
|  |  | Men | 4.2 | 6.7 | -2.5 | 92.5 | 2.5 | 5.0 | 4.2 | 3.3 | 0.8 | 95.8 | 2.5 | 1.7 | 3.3 | 6.7 | -3.3 | 91.7 | 2.5 | 5.8 |
|  |  | Total | 3.9 | 7.1 | -3.2 | 91.8 | 2.5 | 5.7 | 3.9 | 3.9 | 0 | 95.7 | 2.1 | 2.1 | 3.9 | 7.1 | -3.2* | 93.2 | 1.8 | 5.0 |
| 25 | Fluid milk | Women | 6.2 | 6.2 | 0 | 98.8 | 0.6 | 0.6 | 6.2 | 5.0 | 1.2 | 98.8 | 1.2 | 0 | 5.0 | 6.2 | -1.2 | 98.8 | 0 | 1.2 |
|  |  | Men | 15.0 | 7.5 | 7.5* | 90.8 | 8.3 | 0.8 | 15.0 | 9.2 | 5.8 | 90.8 | 7.5 | 1.7 | 9.2 | 7.5 | 1.7 | 96.7 | 2.5 | 0.8 |
|  |  | Total | 10.0 | 6.8 | 3.2* | 95.4 | 3.9 | 0.7 | 10.0 | 6.8 | 3.2* | 95.4 | 3.9 | 0.7 | 6.8 | 6.8 | 0 | 97.9 | 1.1 | 1.1 |
| 26 | Sweet tea / coffee / cocoa | Women | 5.0 | 3.1 | 1.9 | 96.9 | 2.5 | 0.6 | 5.0 | 3.1 | 1.9 | 96.9 | 2.5 | 0.6 | 3.1 | 3.1 | 0 | 100 | 0 | 0 |
|  |  | Men | 7.5 | 7.5 | 0 | 98.3 | 0.8 | 0.8 | 7.5 | 5.8 | 1.7 | 96.7 | 2.5 | 0.8 | 5.8 | 7.5 | -1.7 | 96.7 | 0.8 | 2.5 |
|  |  | Total | 6.0 | 5.0 | 1.1 | 97.5 | 1.8 | 0.7 | 6.0 | 4.3 | 1.8 | 96.8 | 2.5 | 0.7 | 4.3 | 5.0 | -0.7 | 98.6 | 0.4 | 1.1 |
| 27 | Fruit juice and fruit-flavored drinks | Women | 4.3 | 1.2 | 3.1 | 96.9 | 3.1 | 0 | 4.3 | 1.9 | 2.5 | 96.3 | 3.1 | 0.6 | 1.9 | 1.2 | 0.6 | 99.4 | 0.6 | 0 |
|  |  | Men | 5.8 | 4.2 | 1.7 | 95.0 | 3.3 | 1.7 | 5.8 | 1.7 | 4.2 | 95.8 | 4.2 | 0 | 1.7 | 4.2 | -2.5 | 97.5 | 0 | 2.5 |
|  |  | Total | 5.0 | 2.5 | 2.5 | 96.0 | 3.2 | 0.7 | 5.0 | 1.8 | 3.2* | 96.1 | 3.6 | 0.4 | 1.8 | 2.5 | -0.7 | 98.6 | 0.4 | 1.1 |

24hR, multi-pass 24-hour open recall excluding food items consumed in amounts <15g except including <15g for small dried fish; Diff, percentage point difference in population prevalence of food group consumption between DQQ and 24hR (DQQ minus 24hR) or DQQ and OWFR (DQQ minus OWFR); DQQ, Diet Quality Questionnaire; FN, false negatives; FP, false positives; PA, percent agreement coefficient; OWFR, observed weighed food records excluding food items consumed in amounts <15g except including <15g for small dried fish.

^1^ Women n=161; men n=120; total n=281.

* Proportional difference (McNemar test p<0.05) population-prevalence. ** Proportional difference p<0.05 and >10 percentage points, or overreporting (FP) or underreporting (FN) >10%.

Continued.

**Supplementary material**

**Continued, Table 7**

| **Food group** | | **Population^1^** | **DQQ-OWFR** | | | | | | **DQQ-24hR** | | | | | | **24hR-OWFR** | | | | | |
| --- | --- | --- | --- | --- | --- | --- | --- | --- | --- | --- | --- | --- | --- | --- | --- | --- | --- | --- | --- | --- |
|  |  |  | Population-consumption- prevalence (%) | | | Agreement | | | Population-consumption- prevalence (%) | | | Agreement | | | Population-consumption- prevalence (%) | | | Agreement | | |
|  |  |  |  |  |  |  | Misreporting (%) | |  |  |  |  | Misreporting  (%) | |  |  |  |  | Misreporting (%) | |
|  |  |  | DQQ | OWFR | Diff | PA | FP | FN | DQQ | 24hR | Diff | PA | FP | FN | 24hR | OWFR | Diff | PA | FP | FN |
| 28 | Sugar-sweetened beverages (soft drinks) | Women | 1.2 | 0.6 | 0.6 | 99.4 | 0.6 | 0 | 1.2 | 1.2 | 0 | 98.8 | 0.6 | 0.6 | 1.2 | 0.6 | 0.6 | 99.4 | 0.6 | 0 |
|  |  | Men | 10.0 | 5.8 | 4.2 | 92.5 | 5.8 | 1.7 | 10.0 | 7.5 | 2.5 | 92.5 | 5.0 | 2.5 | 7.5 | 5.8 | 1.7 | 96.7 | 2.5 | 0.8 |
|  |  | Total | 5.0 | 2.8 | 2.1 | 96.4 | 2.8 | 0.7 | 5.0 | 3.9 | 1.1 | 96.1 | 2.5 | 1.4 | 3.9 | 2.8 | 1.1 | 98.2 | 1.4 | 0.4 |
| 29 | Fast food | Women | 0.6 | 0 | 0.6 | 99.4 | 0.6 | 0 | 0.6 | 0 | 0.6 | 99.4 | 0.6 | 0 | 0 | 0 | 0 | 100 | 0 | 0 |
|  |  | Men | 0.8 | 0 | 0.8 | 99.2 | 0.8 | 0 | 0.8 | 0 | 0.8 | 99.2 | 0.8 | 0 | 0 | 0 | 0 | 100 | 0 | 0 |
|  |  | Total | 0.7 | 0 | 0.7 | 99.3 | 0.7 | 0 | 0.7 | 0 | 0.7 | 99.3 | 0.7 | 0 | 0 | 0 | 0 | 100 | 0 | 0 |
| *Total food groups*  *(mean (SD))* | | *Women* | *NR* | *NR* | *0.3 (6.8)* | *92.5 (9.0)* | *3.9 (5.8)* | *3.6 (5.6)* | *NR* | *NR* | *1.6 (8.0)* | *92.4 (9.7)* | *4.6 (7.3)* | *3.0 (5.1)* | *NR* | *NR* | *-1.2 (2.6)* | *97.0 (4.8)* | *0.9 (1.3)* | *2.1 (3.6)* |
|  |  | *Men* | *NR* | *NR* | *1.5 (6.2)* | *92.7 (7.8)* | *4.4 (5.3)* | *2.9 (4.7)* | *NR* | *NR* | *2.2 (6.7)* | *92.9 (8.7)* | *4.6 (6.3)* | *2.5 (4.5)* | *NR* | *NR* | *-0.6 (2.6)* | *93.5 (17.8)* | *1.3 (1.6)* | *1.9 (2.9)* |
|  |  | *Total* | *NR* | *NR* | *0.9 (6.4)* | *92.6 (8.4)* | *4.1 (5.4)* | *3.3 (5.1)* | *NR* | *NR* | *1.8 (7.4)* | *92.6 (9.2)* | *4.6 (6.8)* | *2.8 (4.8)* | *NR* | *NR* | *-1.0 (2.5)* | *96.9 (4.3)* | *1.1 (1.2)* | *2.1 (3.3)* |

24hR, multi-pass 24-hour open recall excluding food items consumed in amounts <15g except including <15g for small dried fish; Diff, percentage point difference in population prevalence of food group consumption between DQQ and 24hR (DQQ minus 24hR) or DQQ and OWFR (DQQ minus OWFR); DQQ, Diet Quality Questionnaire; FN, false negatives; FP, false positives; NR, not relevant; PA, percent agreement coefficient; SD, standard deviation; OWFR, observed weighed food records excluding food items consumed in amounts <15g except including <15g for small dried fish.

^1^ Women n=161; men n=120; total n=281.

* Proportional difference (McNemar test p<0.05) population-prevalence. ** Proportional difference p<0.05 and >10 percentage points, or overreporting (FP) or underreporting (FN) >10%.

**Supplementary material**

**Table 8.** Comparison between DQQ-OWFR, DQQ-24hR, and 24hR-OWFR for collecting population-level MDD-W food group consumption data among adult women and men in Northern Rwanda, **excluding food items consumed in amounts <15g except including <15g for small dried fish in OWFR and 24hR**.

| **MDD-W food group** | | **Population^1^** | **DQQ-OWFR** | | | | | | **DQQ-24hR** | | | | | | **24hR-OWFR** | | | | | |
| --- | --- | --- | --- | --- | --- | --- | --- | --- | --- | --- | --- | --- | --- | --- | --- | --- | --- | --- | --- | --- |
|  |  |  | Population-consumption- prevalence (%) | | | Agreement | | | Population-consumption- prevalence (%) | | | Agreement | | | Population-consumption- prevalence (%) | | | Agreement | | |
|  |  |  |  |  |  |  | Misreporting (%) | |  |  |  |  | Misreporting  (%) | |  |  |  |  | Misreporting (%) | |
|  |  |  | DQQ | OWFR | Diff | PA | FP | FN | DQQ | 24hR | Diff | PA | FP | FN | 24hR | OWFR | Diff | PA | FP | FN |
| 1 | Grains, white roots and tubers, and plantains | Women | 95.7 | 100 | -4.3 | 95.7 | 0 | 4.3 | 95.7 | 99.4 | -3.7 | 95.0 | 0.6 | 4.3 | 99.4 | 100 | -0.6 | 99.4 | 0 | 0.6 |
|  |  | Men | 94.2 | 99.2 | -5.0* | 95.0 | 0 | 5.0 | 94.2 | 99.2 | -5.0* | 95.0 | 0 | 5.0 | 99.2 | 99.2 | 0 | 100 | 0 | 0 |
|  |  | Total | 95.0 | 99.6 | -4.6* | 95.4 | 0 | 4.6 | 95.0 | 99.3 | -4.3* | 95.0 | 0.4 | 4.6 | 99.3 | 99.6 | -0.4 | 99.6 | 0 | 0.4 |
| 2 | Pulses (beans, peas and lentils) | Women | 82.6 | 88.8 | -6.2 | 85.1 | 4.3 | 10.6** | 82.6 | 88.2 | -5.6 | 88.2 | 3.1 | 8.7 | 88.2 | 88.8 | -0.6 | 94.4 | 2.5 | 3.1 |
|  |  | Men | 89.2 | 97.5 | -8.3* | 91.7 | 0 | 8.3 | 89.2 | 95.0 | -5.8 | 90.8 | 1.7 | 7.5 | 95.0 | 97.5 | -2.5 | 97.5 | 0 | 2.5 |
|  |  | Total | 85.4 | 92.5 | -7.1* | 87.9 | 2.5 | 9.6 | 85.4 | 91.1 | -5.7* | 89.3 | 2.5 | 8.2 | 91.1 | 92.5 | -1.4 | 95.7 | 1.4 | 2.8 |
| 3 | Nuts and seeds | Women | 45.3 | 20.5 | 24.8** | 66.5 | 29.2** | 4.3 | 45.3 | 9.9 | 35.4** | 62.1 | 36.6** | 1.2 | 9.9 | 20.5 | -10.6** | 80.7 | 4.3 | 14.9** |
|  |  | Men | 40.0 | 20.8 | 19.2** | 70.8 | 24.2** | 5.0 | 40.0 | 10.8 | 29.2** | 65.8 | 31.7** | 2.5 | 10.8 | 20.8 | -10.0* | 88.3 | 0.8 | 10.8** |
|  |  | Total | 43.1 | 20.6 | 22.4** | 68.3 | 27.0** | 4.6 | 43.1 | 10.3 | 32.7** | 63.7 | 34.5** | 1.8 | 10.3 | 20.6 | -10.3** | 84.0 | 2.8 | 13.2** |
| 4 | Dairy | Women | 6.8 | 6.8 | 0 | 98.8 | 0.6 | 0.6 | 6.8 | 6.2 | 0.6 | 98.1 | 1.2 | 0.6 | 6.2 | 6.8 | -0.6 | 98.1 | 0.6 | 1.2 |
|  |  | Men | 19.2 | 10.8 | 8.3* | 90.0 | 9.2 | 0.8 | 19.2 | 12.5 | 6.7 | 91.7 | 7.5 | 0.8 | 12.5 | 10.8 | 1.7 | 98.3 | 1.7 | 0 |
|  |  | Total | 12.1 | 8.5 | 3.6* | 95.0 | 4.3 | 0.7 | 12.1 | 8.9 | 3.2* | 95.4 | 3.9 | 0.7 | 8.9 | 8.5 | 0.4 | 98.2 | 1.1 | 0.7 |
| 5 | Meat, poultry and fish | Women | 31.7 | 38.5 | -6.8* | 88.2 | 2.5 | 9.3 | 31.7 | 36.6 | -5.0 | 87.6 | 3.7 | 8.7 | 36.6 | 38.5 | -1.9 | 98.1 | 0 | 1.9 |
|  |  | Men | 33.3 | 31.7 | 1.7 | 88.3 | 6.7 | 5.0 | 33.3 | 33.3 | 0 | 90.0 | 5.0 | 5.0 | 33.3 | 31.7 | 1.7 | 98.3 | 1.7 | 0 |
|  |  | Total | 32.4 | 35.6 | -3.2 | 88.3 | 4.3 | 7.5 | 32.4 | 35.2 | -2.8 | 88.6 | 4.3 | 7.1 | 35.2 | 35.6 | -0.4 | 98.2 | 0.7 | 1.1 |
| 6 | Eggs | Women | 3.1 | 2.5 | 0.6 | 99.4 | 0.6 | 0 | 3.1 | 1.9 | 1.2 | 98.8 | 1.2 | 0 | 1.9 | 2.5 | -0.6 | 99.4 | 0 | 0.6 |
|  |  | Men | 5.8 | 3.3 | 2.5 | 95.8 | 3.3 | 0.8 | 5.8 | 3.3 | 2.5 | 95.8 | 3.3 | 0.8 | 3.3 | 3.3 | 0 | 100 | 0 | 0 |
|  |  | Total | 4.3 | 2.8 | 1.4 | 97.9 | 1.8 | 0.4 | 4.3 | 2.5 | 1.8 | 97.5 | 2.1 | 0.4 | 2.5 | 2.8 | -0.4 | 99.6 | 0 | 0.4 |

24hR, multi-pass 24-hour open recall excluding food items consumed in amounts <15g except including <15g for small dried fish; Diff, percentage point difference in population prevalence of food group consumption between DQQ and 24hR (DQQ minus 24hR) or DQQ and OWFR (DQQ minus OWFR); DQQ, Diet Quality Questionnaire; FN, false negatives; FP, false positives; MDD-W, Minimum Dietary Diversity for Women; PA, percent agreement coefficient; OWFR, observed weighed food records excluding food items consumed in amounts <15g except including <15g for small dried fish.

^1^ Women n=161; men n=120; total n=281.

* Proportional difference (McNemar test p<0.05) population-prevalence. ** Proportional difference p<0.05 and >10 percentage points, or overreporting (FP) or underreporting (FN) >10%.

Continued.

**Supplementary material**

**Continued, Table 8**

| **MDD-W food group** | | **Population^1^** | **DQQ-OWFR** | | | | | | **DQQ-24hR** | | | | | | **24hR-OWFR** | | | | | |
| --- | --- | --- | --- | --- | --- | --- | --- | --- | --- | --- | --- | --- | --- | --- | --- | --- | --- | --- | --- | --- |
|  |  |  | Population-consumption- prevalence (%) | | | Agreement | | | Population-consumption- prevalence (%) | | | Agreement | | | Population-consumption- prevalence (%) | | | Agreement | | |
|  |  |  |  |  |  |  | Misreporting (%) | |  |  |  |  | Misreporting  (%) | |  |  |  |  | Misreporting (%) | |
|  |  |  | DQQ | OWFR | Diff | PA | FP | FN | DQQ | 24hR | Diff | PA | FP | FN | 24hR | OWFR | Diff | PA | FP | FN |
| 7 | Dark green leafy vegetables | Women | 62.7 | 62.1 | 0.6 | 84.5 | 8.1 | 7.5 | 62.7 | 53.4 | 9.3* | 77.0 | 16.1** | 6.8 | 53.4 | 62.1 | -8.7* | 85.1 | 3.1 | 11.8** |
|  |  | Men | 61.7 | 60.8 | 0.8 | 89.2 | 5.8 | 5.0 | 61.7 | 55.8 | 5.8 | 84.2 | 10.8** | 5.0 | 55.8 | 60.8 | -5.0 | 86.7 | 4.2 | 9.2 |
|  |  | Total | 62.3 | 61.6 | 0.7 | 86.5 | 7.1 | 6.4 | 62.3 | 54.4 | 7.8* | 80.1 | 13.9** | 6.1 | 54.4 | 61.6 | -7.1* | 85.8 | 3.6 | 10.7** |
| 8 | Other vitamin A-rich fruits and vegetables | Women | 26.7 | 16.8 | 9.9* | 82.6 | 13.7** | 3.7 | 26.7 | 17.4 | 9.3* | 84.5 | 12.4** | 3.1 | 17.4 | 16.8 | 0.6 | 94.4 | 3.1 | 2.5 |
|  |  | Men | 25.8 | 16.7 | 9.2* | 84.2 | 12.5** | 3.3 | 25.8 | 17.5 | 8.3* | 86.7 | 10.8** | 2.5 | 17.5 | 16.7 | 0.8 | 94.2 | 3.3 | 2.5 |
|  |  | Total | 26.3 | 16.7 | 9.6* | 83.3 | 13.2** | 3.6 | 26.3 | 17.4 | 8.9* | 85.4 | 11.7** | 2.8 | 17.4 | 16.7 | 0.7 | 94.3 | 3.2 | 2.5 |
| 9 | Other vegetables | Women | 56.5 | 73.9 | -17.4** | 71.4 | 5.6 | 23.0** | 56.5 | 70.8 | -14.3** | 70.8 | 7.5 | 21.7** | 70.8 | 73.9 | -3.1 | 94.4 | 1.2 | 4.3 |
|  |  | Men | 45.8 | 60.8 | -15.0** | 71.7 | 6.7 | 21.7** | 45.8 | 56.7 | -10.8** | 67.5 | 10.8** | 21.7** | 56.7 | 60.8 | -4.2 | 90.8 | 2.5 | 6.7 |
|  |  | Total | 52.0 | 68.3 | -16.4** | 71.5 | 6.0 | 22.4** | 52.0 | 64.8 | -12.8** | 69.4 | 8.9 | 21.7** | 64.8 | 68.3 | -3.6* | 92.9 | 1.8 | 5.3 |
| 10 | Other fruits | Women | 20.5 | 12.4 | 8.1* | 87.0 | 10.6** | 2.5 | 20.5 | 11.8 | 8.7* | 88.8 | 9.9 | 1.2 | 11.8 | 12.4 | -0.6 | 90.7 | 4.3 | 5.0 |
|  |  | Men | 28.3 | 18.3 | 10.0* | 83.3 | 13.3** | 3.3 | 28.3 | 19.2 | 9.2* | 89.2 | 10.0 | 0.8 | 19.2 | 18.3 | 0.8 | 92.5 | 4.2 | 3.3 |
|  |  | Total | 23.8 | 14.9 | 8.9* | 85.4 | 11.7** | 2.8 | 23.8 | 14.9 | 8.9* | 89.0 | 10.0 | 1.1 | 14.9 | 14.9 | 0 | 91.5 | 4.3 | 4.3 |
| *Total food groups*  *(mean (SD))* | | *Women* | *NR* | *NR* | *0.9 (11.4)* | *85.9 (10.8)* | *7.5 (8.9)* | *6.6 (6.7)* | *NR* | *NR* | *3.6 (13.5)* | *85.1 (11.9)* | *9.2 (11.0)* | *5.6 (6.5)* | *NR* | *NR* | *-1.7 (2.7)* | *95.2 (4.5)* | *1.5 (1.6)* | *3.2 (3.4)* |
|  |  | *Men* | *NR* | *NR* | *2.3 (10.0)* | *86.0 (8.7)* | *8.2 (7.2)* | *5.8 (6.0)* | *NR* | *NR* | *4.0 (11.1)* | *85.7 (10.6)* | *9.2 (8.9)* | *5.2 (6.2)* | *NR* | *NR* | *-0.5 (2.5)* | *95.7 (4.4)* | *1.9 (1.6)* | *2.4 (3.2)* |
|  |  | *Total* | *NR* | *NR* | *1.5 (10.6)* | *86.0 (9.7)* | *7.8 (7.9)* | *6.3 (6.4)* | *NR* | *NR* | *3.8 (12.4)* | *85.3 (11.2)* | *9.2 (10.0)* | *5.5 (6.3)* | *NR* | *NR* | *-2.3 (3.7)* | *94.0 (5.5)* | *1.9 (1.5)* | *4.1 (4.5)* |

24hR, multi-pass 24-hour open recall excluding food items consumed in amounts <15g except including <15g for small dried fish; Diff, percentage point difference in population prevalence of food group consumption between DQQ and 24hR (DQQ minus 24hR) or DQQ and OWFR (DQQ minus OWFR); DQQ, Diet Quality Questionnaire; FN, false negatives; FP, false positives; MDD-W, Minimum Dietary Diversity for Women; NR, not relevant; PA, percent agreement coefficient; SD, standard deviation; OWFR, observed weighed food records excluding food items consumed in amounts <15g except including <15g for small dried fish.

^1^ Women n=161; men n=120; total n=281.

* Proportional difference (McNemar test p<0.05) population-prevalence. ** Proportional difference p<0.05 and >10 percentage points, or overreporting (FP) or underreporting (FN) >10%.

**Supplementary material**

**Table 9.** Comparison between DQQ-OWFR, DQQ-24hR, and 24hR-OWFR for achieving binary indicators MDD-W, All-5, HDI, PFC, and UFC among adult women and men in Northern Rwanda, **excluding food items consumed in amounts <15g except including <15g for small dried fish in OWFR and 24hR**.

| **Indicator**  **(%)** | **Population^1^** | **DQQ-OWFR** | | | | | | **DQQ-24hR** | | | | | | **24hR-OWFR** | | | | | |
| --- | --- | --- | --- | --- | --- | --- | --- | --- | --- | --- | --- | --- | --- | --- | --- | --- | --- | --- | --- |
|  |  | Population-  prevalence (%) | | | Agreement | | | Population-  prevalence (%) | | | Agreement | | | Population-  prevalence (%) | | | Agreement | | |
|  |  |  |  |  |  | Misreporting (%) | |  |  |  |  | Misreporting (%) | |  |  |  |  | Misreporting (%) | |
|  |  | DQQ | OWFR | Diff | PA | FP | FN | DQQ | 24hR | Diff | PA | FP | FN | 24hR | OWFR | Diff | PA | FP | FN |
| MDD-W | Women | 46.0 | 40.4 | 5.6 | 74.5 | 15.5** | 9.9 | 46.0 | 29.8 | 16.1** | 71.4 | 22.4** | 6.2 | 29.8 | 40.4 | -10.6** | 83.2 | 3.1 | 13.7** |
|  | Men | 45.0 | 38.3 | 6.7 | 76.7 | 15.0** | 8.3 | 45.0 | 35.0 | 10.0* | 76.7 | 16.7** | 6.7 | 35.0 | 38.3 | -3.3 | 91.7 | 2.5 | 5.8 |
|  | Total | 45.6 | 39.5 | 6.0 | 75.4 | 15.3** | 9.3 | 45.6 | 32.0 | 13.5** | 73.7 | 19.9** | 6.4 | 32.0 | 39.5 | -7.5* | 86.8 | 2.8 | 10.3** |
| All-5 | Women | 11.2 | 4.3 | 6.8 | 90.7 | 8.1 | 1.2 | 11.2 | 8.1 | 3.1 | 93.2 | 5.0 | 1.9 | 8.1 | 4.3 | 3.7 | 93.8 | 5.0 | 1.2 |
|  | Men | 14.2 | 8.3 | 5.8 | 85.8 | 10.0 | 4.2 | 14.2 | 11.7 | 2.5 | 90.8 | 5.8 | 3.3 | 11.7 | 8.3 | 3.3 | 93.3 | 5.0 | 1.7 |
|  | Total | 12.5 | 6.0 | 6.4* | 88.6 | 8.9 | 2.5 | 12.5 | 9.6 | 2.8 | 92.2 | 5.3 | 2.5 | 9.6 | 6.0 | 3.6* | 93.6 | 5.0 | 1.4 |
| PFC | Women | 21.7 | 13.7 | 8.1* | 85.7 | 11.2** | 3.1 | 21.7 | 13.0 | 8.7* | 86.3 | 11.2** | 2.5 | 13.0 | 13.7 | -0.6 | 90.7 | 4.3 | 5.0 |
|  | Men | 27.5 | 17.5 | 10.0* | 78.3 | 15.8** | 5.8 | 27.5 | 21.7 | 5.8 | 85.8 | 10.0 | 4.2 | 21.7 | 17.5 | 4.2 | 87.5 | 8.3 | 4.2 |
|  | Total | 24.2 | 15.3 | 8.9* | 82.6 | 13.2** | 4.3 | 24.2 | 16.7 | 7.5* | 86.1 | 10.7** | 3.2 | 16.7 | 15.3 | 1.4 | 89.3 | 6.0 | 4.6 |
| UFC | Women | 4.3 | 0.6 | 3.7* | 96.3 | 3.7 | 0 | 4.3 | 1.2 | 3.1 | 95.7 | 3.7 | 0.6 | 1.2 | 0.6 | 0.6 | 99.4 | 0.6 | 0 |
|  | Men | 10.8 | 6.1 | 4.2 | 92.5 | 5.8 | 1.7 | 10.8 | 8.3 | 2.5 | 92.5 | 5.0 | 2.5 | 8.3 | 6.1 | 1.7 | 96.7 | 2.5 | 0.8 |
|  | Total | 7.1 | 3.2 | 3.9* | 94.7 | 4.6 | 0.7 | 7.1 | 4.3 | 2.8 | 94.3 | 4.3 | 1.4 | 4.3 | 3.2 | 1.1 | 98.2 | 1.4 | 0.4 |
| HDI | Women | 19.9 | 13.7 | 6.2 | 86.3 | 9.9 | 3.7 | 19.9 | 13.0 | 6.8* | 87.0 | 9.9 | 3.1 | 13.0 | 13.7 | -0.6 | 90.7 | 4.3 | 5.0 |
|  | Men | 23.3 | 15.8 | 7.5 | 79.2 | 14.2** | 6.7 | 23.3 | 19.2 | 4.2 | 85.8 | 9.2 | 5.0 | 19.2 | 15.8 | 3.3 | 88.3 | 7.5 | 4.2 |
|  | Total | 21.4 | 14.6 | 6.8* | 83.3 | 11.7** | 5.0 | 21.4 | 15.7 | 5.7* | 86.5 | 9.6 | 3.9 | 15.7 | 14.6 | 1.1 | 89.7 | 5.7 | 4.6 |

24hR, multi-pass 24-hour open recall excluding food items consumed in amounts <15g except including <15g for small dried fish; Diff, percentage point difference in population prevalence of food group consumption between DQQ and 24hR (DQQ minus 24hR), between DQQ and OWFR (DQQ minus OWFR), or between 24hR and OWFR (24hR minus OWFR); DQQ, Diet Quality Questionnaire; FN, false negatives; FP, false positives; HDI, Healthy Diet Indicator; MDD-W, Minimum Dietary Diversity for Women; PA, percent agreement coefficient; PFC, Protective Food Consumption; UFC, Unhealthy Food Consumption; OWFR, observed weighed food records excluding food items consumed in amounts <15g except including <15g for small dried fish.

^1^ Women n=161; men n=120; total n=281.

* Proportional difference (McNemar test p<0.05) population-prevalence.

** Proportional difference p<0.05 and >10 percentage points, or overreporting (FP) or underreporting (FN) >10%.

**Supplementary material**

**Table 10.** Comparison between DQQ-OWFR, DQQ-24hR, and 24hR-OWFR of FGDS, NCD-Protect, and NCD-Risk scores among adult women and men in Northern Rwanda, **excluding food items consumed in amounts <15g except including <15g for small dried fish in OWFR and 24hR**.

|  |  | **DQQ-OWFR** | | | | | **DQQ-24hR** | | | | | **24hR-OWFR** | | | | |
| --- | --- | --- | --- | --- | --- | --- | --- | --- | --- | --- | --- | --- | --- | --- | --- | --- |
| **Indicator^1^** | **Population^2^** | DQQ^3^ | OWFR^3^ | Diff  in mean | Diff  in % | p | DQQ^3^ | 24hR^3^ | Diff  in mean | Diff  in % | p | 24hR^3^ | OWFR^3^ | Diff  in mean | Diff  in % | p |
| FGDS | Women | 4.3 (1.8) | 4.2 (1.3) | 0.1 | 0.9 | 0.392 | 4.3 (1.8) | 4.0 (1.3) | 0.4 | 3.6 | 0.003 | 4.0 (1.3) | 4.2 (1.3) | -0.3 | -2.7 | 0.001 |
|  | Men | 4.4 (1.8) | 4.2 (1.5) | 0.2 | 2.3 | 0.056 | 4.4 (1.8) | 4.0 (1.5) | 0.4 | 4.0 | 0.002 | 4.0 (1.5) | 4.2 (1.5) | -0.2 | -1.7 | 0.023 |
|  | Total | 4.4 (1.8) | 4.2 (1.4) | 0.2 | 1.5 | 0.059 | 4.4 (1.8) | 4.0 (1.4) | 0.4 | 3.8 | <0.001 | 4.0 (1.4) | 4.2 (1.4) | -0.2 | -2.2 | <0.001 |
| NCD-Protect | Women | 3.4 (1.5) | 3.3 (1.1) | 0.2 | 2.1 | 0.088 | 3.4 (1.5) | 3.0 (1.2) | 0.5 | 5.2 | <0.001 | 3.0 (1.2) | 3.3 (1.1) | -0.3 | -3.1 | <0.001 |
|  | Men | 3.3 (1.6) | 3.1 (1.3) | 0.2 | 2.3 | 0.043 | 3.3 (1.6) | 2.9 (1.3) | 0.4 | 4.4 | <0.001 | 2.9 (1.3) | 3.1 (1.3) | -0.2 | -2.1 | 0.019 |
|  | Total | 3.4 (1.5) | 3.2 (1.2) | 0.2 | 2.2 | 0.010 | 3.4 (1.5) | 3.0 (1.3) | 0.4 | 4.9 | <0.001 | 3.0 (1.3) | 3.2 (1.2) | -0.2 | -2.7 | <0.001 |
| NCD-Risk | Women | 0 (0 – 0) | 0 (0 – 0) | - | - | 0.461 | 0 (0 – 0) | 0 (0 – 0) | - | - | 0.053 | 0 (0 – 0) | 0 (0 – 0) | - | - | 0.132 |
|  | Men | 0 (0 – 0) | 0 (0 – 0) | - | - | 0.033 | 0 (0 – 0) | 0 (0 – 0) | - | - | 0.011 | 0 (0 – 0) | 0 (0 – 0) | - | - | 0.806 |
|  | Total | 0 (0 – 0) | 0 (0 – 0) | - | - | 0.045 | 0 (0 – 0) | 0 (0 – 0) | - | - | 0.002 | 0 (0 – 0) | 0 (0 – 0) | - | - | 0.491 |

24hR, multi-pass 24-hour open recall excluding food items consumed in amounts <15g except including <15g for small dried fish; Diff in mean, mean difference in indicator scores between DQQ and 24hR (DQQ minus 24hR), or DQQ and OWFR (DQQ minus OWFR), or 24hR and OWFR (24hR minus OWFR); Diff in pp, percentage point difference of indicator scores between DQQ and 24hR (DQQ minus 24hR), or DQQ and OWFR (DQQ minus OWFR), or 24hR and OWFR (24hR minus OWFR); DQQ, Diet Quality Questionnaire; FGDS, Food Group Diversity Score; NCD, non-communicable diseases; p, p-value of paired samples t-test for FGDS and NCD-Protect, and of Wilcoxon signed-rank test for NCD-Risk; OWFR, observed weighed food record excluding food items consumed in amounts <15g except including <15g for small dried fish.

^1^ Scoring range FGDS: 0 – 10; NCD-Protect: 0 – 9; NCD-Risk: 0 – 9.

^2^ Women n=161; men n=120; total n=281.

^3^ Values are in mean (SD) for FGDS and NCD-Protect, and in median (25^th^ – 75^th^ percentiles) for NCD-Risk.

**Supplementary material**

**Table 11.** Correlation and validity coefficients for diet quality indicator scores calculated from DQQ, 24hR, and OWFR of adult women and men in Northern Rwanda.

| **Pearson’s correlation coefficient (95% CI)** | **Population^1^** | **FGDS** | **NCD-Protect** | **NCD-Risk** |
| --- | --- | --- | --- | --- |
| *r*DQQ-OWFR | Women | 0.63 (0.53, 0.72) | 0.50 (0.37, 0.61) | 0.19 (0.03, 0.33) |
|  | Men | 0.68 (0.57, 0.77) | 0.72 (0.62, 0.80) | 0.54 (0.40, 0.66) |
|  | Total | 0.65 (0.58, 0.71) | 0.60 (0.52, 0.67) | 0.39 (0.28, 0.48) |
| *r*DQQ-24hR | Women | 0.55 (0.43, 0.65) | 0.45 (0.32, 0.57) | 0.44 (0.31, 0.56) |
|  | Men | 0.64 (0.52, 0.74) | 0.69 (0.58, 0.77) | 0.76 (0.67, 0.83) |
|  | Total | 0.59 (0.51, 0.66) | 0.56 (0.47, 0.64) | 0.64 (0.57, 0.71) |
| *r*24hR-OWFR | Women | 0.71 (0.63, 0.78) | 0.71 (0.63, 0.78) | 0.60 (0.49, 0.69) |
|  | Men | 0.86 (0.80, 0.90) | 0.78 (0.70, 0.84) | 0.58 (0.45, 0.69) |
|  | Total | 0.78 (0.74, 0.83) | 0.74 (0.69, 0.78) | 0.58 (0.50, 0.65) |
| **Validity coefficient^2^** |  |  |  |  |
| ρDQQT | Women | 0.70^2^ | 0.56^2^ | 0.37^2^ |
|  | Men | 0.71^3^ | 0.80^3^ | 0.84^3^ |
|  | Total | 0.70^2^ | 0.67^2^ | 0.66^2^ |
| ρ24hRT | Women | 0.79^3^ | 0.80^3^ | 1.18^4^ |
|  | Men | 0.90^3^ | 0.86^3^ | 0.90^3^ |
|  | Total | 0.84^3^ | 0.83^3^ | 0.98^3^ |
| ρOWFRT | Women | 0.90^3^ | 0.89^3^ | 0.51^2^ |
|  | Men | 0.96^3^ | 0.90^3^ | 0.64^2^ |
|  | Total | 0.93^3^ | 0.89^3^ | 0.59^2^ |

24hR, multi-pass 24-hour open recall excluding food items consumed in amounts <15g except including <15g for small dried fish; CI, confidence intervals; DQQ, Diet Quality Questionnaire; FGDS, Food Group Diversity Score; NCD, non-communicable diseases; OWFR, observed weighed food record excluding food items consumed in amounts <15g except including <15g for small dried fish; ρ, validity coefficient in relation to T; r, Pearson correlation coefficient.

^1^ Women n=161; men n=120; total n=281.

^2^ Validity coefficient moderate: 0.30 – 0.70 (35).

^3^ Validity coefficient high: >0.70 (35).

^4^ Heywood case (validity coefficient >1), i.e., biased validity coefficient likely due to correlated random errors between DQQ, OWFR, and 24hR, and the low correlation between DQQ-OWFR (35).

**Supplementary material**

**Table 12a.** Correlation and validity coefficients of FGDS, NCD-Protect, and NCD-Risk estimated from DQQ, 24hR, and OWFR of adults in Northern Rwanda (n=281).

| **Pearson’s correlation coefficient (95% CI)** | **FGDS** | **NCD-Protect** | **NCD-Risk** |
| --- | --- | --- | --- |
| *r*DQQ-OWFR | 0.65 (0.58, 0.71) | 0.60 (0.52, 0.67) | 0.39 (0.28, 0.48) |
| *r*DQQ-24hR | 0.59 (0.51, 0.66) | 0.56 (0.47, 0.64) | 0.64 (0.57, 0.71) |
| *r*24hR-OWFR | 0.78 (0.74, 0.83) | 0.74 (0.69, 0.78) | 0.58 (0.50, 0.65) |
| **Validity coefficient^2^** |  |  |  |
| ρDQQT | 0.70^1^ | 0.67^1^ | 0.66^1^ |
| ρ24hRT | 0.84^2^ | 0.83^2^ | 0.98^2^ |
| ρOWFRT | 0.93^2^ | 0.89^2^ | 0.59^1^ |

24hR, multi-pass 24-hour open recall excluding food items consumed in amounts <15g except including <15g for small dried fish; CI, confidence intervals; DQQ, Diet Quality Questionnaire; FGDS, Food Group Diversity Score; NCD, non-communicable diseases; OWFR, observed weighed food record excluding food items consumed in amounts <15g except including <15g for small dried fish; ρ, validity coefficient in relation to T; r, Pearson correlation coefficient.

^1^ Validity coefficient moderate: 0.30 – 0.70 (35).

^2^ Validity coefficient high: >0.70 (35).

**Table 12b.** Correlation coefficients, and validity coefficients calculated **from lowest bound of 95% CI** of correlation coefficients of FGDS, NCD-Protect, and NCD-Risk estimated from DQQ, 24hR, and OWFR of adults in Northern Rwanda (n=281).

| **Pearson’s correlation coefficient (95% CI)** | **FGDS** | **NCD-Protect** | **NCD-Risk** |
| --- | --- | --- | --- |
| *r*DQQ-OWFR | 0.65 (0.58, 0.71) | 0.60 (0.52, 0.67) | 0.39 (0.28, 0.48) |
| *r*DQQ-24hR | 0.59 (0.51, 0.66) | 0.56 (0.47, 0.64) | 0.64 (0.57, 0.71) |
| *r*24hR-OWFR | 0.78 (0.74, 0.83) | 0.74 (0.69, 0.78) | 0.58 (0.50, 0.65) |
| **Validity coefficient^2^** |  |  |  |
| ρDQQT | 0.63^1^ | 0.60^1^ | 0.56^1^ |
| ρ24hRT | 0.81^2^ | 0.79^2^ | 1.01^3^ |
| ρOWFRT | 0.92^2^ | 0.87^2^ | 0.50^1^ |

24hR, multi-pass 24-hour open recall excluding food items consumed in amounts <15g except including <15g for small dried fish; CI, confidence intervals; DQQ, Diet Quality Questionnaire; FGDS, Food Group Diversity Score; NCD, non-communicable diseases; OWFR, observed weighed food record excluding food items consumed in amounts <15g except including <15g for small dried fish; ρ, validity coefficient in relation to T calculated from the lowest bound of 95% CI of the Pearson correlation coefficients; r, Pearson correlation coefficient.

^1^ Validity coefficient moderate: 0.30 – 0.70 (35).

^2^ Validity coefficient high: >0.70 (35).

^3^ Heywood case (validity coefficient >1), i.e., biased validity coefficient likely due to correlated random errors between DQQ, OWFR, and 24hR and the low correlation between DQQ-OWFR (35).

**Supplementary material**

**Table 13.** Comparison between DQQ-OWFR, DQQ-24hR, and 24hR-OWFR for collecting food group consumption data among adults in Northern Rwanda **including food items consumed in less than 15g** (n = 281).

| **Food group** | | **DQQ-24hR** | | | | | | **DQQ-OWFR** | | | | | | **24hR-OWFR** | | | | | |
| --- | --- | --- | --- | --- | --- | --- | --- | --- | --- | --- | --- | --- | --- | --- | --- | --- | --- | --- | --- |
|  |  | Population consumption prevalence (%) | | | Agreement | | | Population  consumption  prevalence (%) | | | Agreement | | | Population  consumption  prevalence (%) | | | Agreement | | |
|  |  |  |  |  |  | Misreporting  (%) | |  |  |  |  | Misreporting (%) | |  |  |  |  | Misreporting (%) | |
|  |  | DQQ | OWFR | Diff | PA | FP | FN | DQQ | 24hR | Diff | PA | FP | FN | 24hR | OWFR | Diff | PA | FP | FN |
| 1 | Foods made from grains | 50.2 | 48.4 | 1.8 | 81.1 | 10.3** | 8.5 | 50.2 | 61.6 | -11.4** | 80.8 | 3.9 | 15.3** | 61.6 | 48.4 | 13.2** | 86.1 | 13.5** | 0.4 |
| 2 | Whole grains | 44.1 | 44.5 | -0.4 | 84.7 | 7.5 | 7.8 | 44.1 | 42.0 | 2.1 | 85.1 | 8.5 | 6.4 | 42.0 | 44.5 | -2.5 | 90.4 | 3.6 | 6.0 |
| 3 | White roots, tubers, and plantains | 81.9 | 93.2 | -11.4** | 87.2 | 0.7 | 12.1** | 81.9 | 92.5 | -10.7** | 86.5 | 1.4 | 12.1** | 92.5 | 93.2 | -0.7 | 97.9 | 0.7 | 1.4 |
| 4 | Legumes | 85.4 | 92.5 | -7.1* | 87.9 | 2.5 | 9.6 | 85.4 | 92.5 | -7.1* | 88.6 | 2.1 | 9.3 | 92.5 | 92.5 | 0 | 95.7 | 2.1 | 2.1 |
| 5 | Vitamin A-rich orange vegetables | 20.6 | 15.7 | 5.0* | 88.6 | 8.2 | 3.2 | 20.6 | 14.9 | 5.7* | 90.0 | 7.5 | 2.1 | 14.9 | 15.7 | -0.7 | 96.4 | 1.4 | 2.1 |
| 6 | Dark green leafy vegetables | 62.3 | 63.0 | -0.7 | 87.2 | 6.0 | 6.8 | 62.3 | 62.6 | -0.4 | 86.8 | 6.4 | 6.8 | 62.6 | 63.0 | -0.4 | 94.7 | 2.5 | 2.8 |
| 7 | Other vegetables | 52.0 | 72.2 | -20.3** | 71.9 | 3.9 | 24.2** | 52.0 | 68.3 | -16.4** | 70.8 | 6.4 | 22.8** | 68.3 | 72.2 | -3.9* | 94.0 | 1.1 | 5.0 |
| 8 | Vitamin A-rich fruits | 7.1 | 2.1 | 5.0* | 92.9 | 6.0 | 1.1 | 7.1 | 3.6 | 3.6* | 95.0 | 4.3 | 0.7 | 3.6 | 2.1 | 1.4 | 97.2 | 2.1 | 0.7 |
| 9 | Citrus | 1.8 | 0.7 | 1.1 | 98.9 | 1.1 | 0 | 1.8 | 0.4 | 1.4 | 98.6 | 1.4 | 0 | 0.4 | 0.7 | -0.4 | 99.6 | 0 | 0.4 |
| 10 | Other fruits | 22.4 | 14.2 | 8.2* | 86.1 | 11.1** | 2.8 | 22.4 | 14.6 | 7.8* | 90.0 | 8.9 | 1.1 | 14.6 | 14.2 | 0.4 | 91.8 | 4.3 | 3.9 |
| 11 | Baked / grain-based sweets | 4.3 | 0.7 | 3.6* | 96.4 | 3.6 | 0 | 4.3 | 1.4 | 2.8* | 97.2 | 2.8 | 0 | 1.4 | 0.7 | 0.7 | 99.3 | 0.7 | 0 |
| 12 | Other sweets | 1.4 | 0.7 | 0.7 | 97.9 | 1.4 | 0.7 | 1.4 | 0.4 | 1.1 | 98.2 | 1.4 | 0.4 | 0.4 | 0.7 | -0.4 | 99.6 | 0 | 0.4 |
| 13 | Eggs | 4.3 | 3.2 | 1.1 | 98.2 | 1.4 | 0.4 | 4.3 | 3.2 | 1.1 | 97.5 | 1.8 | 0.7 | 3.2 | 3.2 | 0 | 99.3 | 0.4 | 0.4 |
| 14 | Cheese | 0 | 0 | 0 | 100 | 0 | 0 | 0 | 0 | 0 | 100 | 0 | 0 | 0 | 0 | 0 | 100 | 0 | 0 |
| 15 | Yogurt | 3.6 | 1.8 | 1.8 | 98.2 | 1.8 | 0 | 3.6 | 2.5 | 1.1 | 98.2 | 1.4 | 0.4 | 2.5 | 1.8 | 0.7 | 99.3 | 0.7 | 0 |
| 16 | Processed meats | 0 | 0 | 0 | 100 | 0 | 0 | 0 | 0 | 0 | 100 | 0 | 0 | 0 | 0 | 0 | 100 | 0 | 0 |
| 17 | Unprocessed red meat (ruminant) | 2.5 | 1.4 | 1.1 | 98.2 | 1.4 | 0.4 | 2.5 | 1.8 | 0.7 | 98.6 | 1.1 | 0.4 | 1.8 | 1.4 | 0.4 | 99.6 | 0.4 | 0 |
| 18 | Unprocessed red meat (non-ruminant) | 0 | 0 | 0 | 100 | 0 | 0 | 0 | 0 | 0 | 100 | 0 | 0 | 0 | 0 | 0 | 100 | 0 | 0 |
| 19 | Poultry | 1.4 | 0.4 | 1.1 | 98.9 | 1.1 | 0 | 1.4 | 0.7 | 0.7 | 99.3 | 0.7 | 0 | 0.7 | 0.4 | 0.4 | 99.6 | 0.4 | 0 |
| 20 | Fish and seafood | 30.6 | 34.5 | -3.9 | 89.0 | 3.6 | 7.5 | 30.6 | 33.8 | -3.2 | 89.0 | 3.9 | 7.1 | 33.8 | 34.5 | -0.7 | 98.6 | 0.4 | 1.1 |

24hR: multi-pass 24-hour open recall; D: Percentage point difference in population prevalence of food group consumption between DQQ and 24hR (DQQ minus 24hR) or DQQ and OWFR (DQQ minus OWFR); DQQ: Diet Quality Questionnaire; FN: False negatives; FP: False positives; NR: not relevant; PA: Percent agreement coefficient. OWFR: observed weighed food records.

* Proportional difference (McNemar test p<0.05) population-prevalence. ** Proportional difference p<0.05 and >10 percentage points, or overreporting (FP) or underreporting (FN) >10%.

Continued.

**Continued, Table 13**

| **Food group** | | **DQQ-24hR** | | | | | | **DQQ-OWFR** | | | | | | **24hR-OWFR** | | | | | |
| --- | --- | --- | --- | --- | --- | --- | --- | --- | --- | --- | --- | --- | --- | --- | --- | --- | --- | --- | --- |
|  |  | Population consumption prevalence (%) | | | Agreement | | | Population  consumption  prevalence (%) | | | Agreement | | | Population  consumption  prevalence (%) | | | Agreement | | |
|  |  |  |  |  |  | Misreporting (%) | |  |  |  |  | Misreporting (%) | |  |  |  |  | Misreporting (%) | |
|  |  | DQQ | OWFR | Diff | PA | FP | FN | DQQ | 24hR | Diff | PA | FP | FN | 24hR | OWFR | Diff | PA | FP | FN |
| 21 | Nuts and seeds | 43.1 | 47.0 | -3.9 | 86.8 | 4.6 | 8.5 | 43.1 | 44.1 | -1.1 | 89.7 | 4.6 | 5.7 | 44.1 | 47.0 | -2.8 | 93.6 | 1.8 | 4.6 |
| 22 | Packaged ultra-processed salty snacks | 0 | 0 | 0 | 100 | 0 | 0 | 0 | 0 | 0 | 100 | 0 | 0 | 0 | 0 | 0 | 100 | 0 | 0 |
| 23 | Instant noodles | 0 | 0 | 0 | 100 | 0 | 0 | 0 | 0 | 0 | 100 | 0 | 0 | 0 | 0 | 0 | 100 | 0 | 0 |
| 24 | Deep fried foods | 3.9 | 7.1 | -3.2 | 91.8 | 2.5 | 5.7 | 3.9 | 3.9 | 0 | 95.7 | 2.1 | 2.1 | 3.9 | 7.1 | -3.2 | 93.2 | 1.8 | 5.0 |
| 25 | Fluid milk | 10.0 | 6.8 | 3.2* | 95.4 | 3.9 | 0.7 | 10.0 | 6.8 | 3.2* | 95.4 | 3.9 | 0.7 | 6.8 | 6.8 | 0 | 97.9 | 1.1 | 1.1 |
| 26 | Sweet tea / coffee / cocoa | 6.0 | 5.0 | 1.1 | 97.5 | 1.8 | 0.7 | 6.0 | 4.3 | 1.8 | 96.8 | 2.5 | 0.7 | 4.3 | 5.0 | -0.7 | 98.6 | 0.4 | 1.1 |
| 27 | Fruit juice and fruit-flavored drinks | 5.0 | 2.5 | 2.5 | 96.1 | 3.2 | 0.7 | 5.0 | 1.8 | 3.2 | 96.1 | 3.6 | 0.4 | 1.8 | 2.5 | -0.7 | 98.6 | 0.4 | 1.1 |
| 28 | Sugar-sweetened beverages  (soft drinks) | 5.0 | 2.8 | 2.1 | 96.4 | 2.8 | 0.7 | 5.0 | 3.9 | 1.1 | 96.1 | 2.5 | 1.4 | 3.9 | 2.8 | 1.1 | 98.2 | 1.4 | 0.4 |
| 29 | Fast food | 0 | 0 | 0 | 100 | 0 | 0 | 0 | 0 | 0 | 100 | 0 | 0 | 0 | 0 | 0 | 100 | 0 | 0 |

24hR: multi-pass 24-hour open recall; Diff: Percentage point difference in population prevalence of food group consumption between DQQ and 24hR (DQQ minus 24hR), between DQQ and OWFR (DQQ minus OWFR), or between 24hR and OWFR (24hR minus OWFR); DQQ: Diet Quality Questionnaire; FN: False negatives; FP: False positives; PA: Percent agreement coefficient. SD: standard deviation; OWFR: observed weighed food records.

* Proportional difference (McNemar test p<0.05) population-prevalence.

** Proportional difference p<0.05 and >10 percentage points, or overreporting (FP) or underreporting (FN) >10%.
